# TRPM2 is a direct pain transducer

**DOI:** 10.1073/pnas.2532289123

**Published:** 2026-09-16

**Authors:** Linda Varghese, Mujahid Alizada, Jinquan Yang, Ye Feng, Xiaoqiu Yuan, Mitali Malhotra, Xuming Zhang

**Affiliations:** ^a^ School of Life Sciences, University of Warwick, Coventry CV4 7AL, United Kingdom

**Keywords:** pain, TRP channels, PGE2, autoantibody, DRG

## Abstract

Chronic pain results from maladaptive interaction between the immune and nervous systems. TRPM2 channels in immune cells (immune TRPM2) are believed to facilitate chronic pain by indirectly promoting immune-inflammatory responses, whereas TRPM2 in sensory neurons (neuronal TRPM2) acts as a warmth sensor critical to sense innocuous warm temperatures. However, neuronal TRPM2 mediates the warmth sensitivity of less than 3.5% of sensory neurons. The functions of the vast majority of TRPM2^+^ neurons are unknown. Here, we show that neuronal TRPM2 functions as a pain sensor responsible for directly transducing acute and chronic pain independently of immune TRPM2. Both chronic arthritis pain and neuropathic pain were markedly reduced in TRPM2-knockout mice, and the pain deficit was recapitulated by sole deletion of neuronal TRPM2. However, immune and inflammatory responses were largely similar between wild-type and neuronal TRPM2-deficient mice. Moreover, antagonizing TRPM2 rapidly reversed arthritis pain and neuropathic pain. Notably, TRPM2 is activated by PGE2 and IgG immune complex (IgG-IC) through coupling to GαoA and FcγRI, respectively, independently of conventional signaling messengers. Consistently, acute pain induced by PGE2 and IgG-IC was abolished in TRPM2 mutant mice. We conclude that neuronal TRPM2 is a convergent direct pain transducer independently of inflammation, representing an appealing target for alleviating chronic pain.

## Significance

TRPM2 channels are expressed in immune inflammatory cells participating in immune inflammation and pain. In this research, we demonstrated that TRPM2 channels in sensory neurons are essential to directly transduce chronic arthritis pain and neuropathic pain with little involvement of TRPM2 in immune inflammatory cells. Deletion of TRPM2 in sensory neurons markedly reduced chronic arthritis pain and neuropathic pain without influencing tissue inflammation and joint pathology. We further revealed that TRPM2 in sensory neurons also directly transduce acute pain evoked by prostaglandin E2 and IgG immune complex, both of which are also markedly increased in chronic arthritis pain and neuropathic pain. TRPM2 in sensory neurons therefore acts as a direct pain sensor critical to both acute and chronic pain.

Neuroimmune interaction is fundamental to chronic pain (1, 2). Activation of either the nociceptive system or immune system mobilizes both systems, triggering cooperative responses for removing pathogens and restoring injured tissues. This interacting process often results in chronic pain. For example, nerve injury causes neuropathic pain, while rheumatoid arthritis—an autoimmune disease—produces arthritis pain. The neuroimmune crosstalk suggests common molecular mechanisms underlying chronic arthritis pain and neuropathic pain. Indeed, recent research reported that autoantibodies underlie both rheumatoid arthritis pain and nerve injury-induced neuropathic pain (3–6). IgG-IC acts by directly exciting nociceptive neurons through binding FcγRI—a high affinity immune receptor for IgG (7)—on sensory DRG neurons, driving arthritis pain and neuropathic pain (3–6, 8, 9). However, how IgG-IC excites sensory neurons remains elusive. Furthermore, it is not known whether other common mechanisms coexist with autoantibodies mediating chronic arthritis pain and neuropathic pain.

TRPM2 is a Ca^2+^-permeable nonselective cation channel mainly expressed in immune cells (immune TRPM2) such as macrophages, monocytes, lymphocytes, and neutrophils (2, 10). It is activated by Ca^2+^ and the cellular metabolite ADPR functioning as a metabolic sensor (2, 11–15). TRPM2 is also a sensor for oxidative stress, though oxidative stress activates TRPM2 indirectly through ADPR produced from the poly-ADPR polymerase (PARP-1)/PAR glycohydrolase (PARG) pathway activated during oxidative stress (2, 16, 17). Functionally, TRPM2 activation by oxidative stress promotes production of chemokine (CXCL2) and cytokines (IL-1β, IL-6, and TNFα) in monocytes/macrophages aggravating neutrophil infiltration and inflammatory response (12, 18–21). It is thus thought that TRPM2 promotes pain indirectly by amplifying immune and inflammatory responses through immune TRPM2 (22, 23). TRPM2 was also found in sensory DRG neurons (neuronal TRPM2) where it acts as a warmth sensor (24). However, the warmth-sensitive DRG neurons attributable to TRPM2 are less than 3.5%, despite that around 37% of DRG neurons expressed functional TRPM2 (24, 25). This raised the question of what the functions of the vast majority of TRPM2^+^ DRG neurons are and whether neuronal TRPM2 acts as a direct pain transducer apart from being a thermo-sensor.

## Results

### Neuronal TRPM2 Is Crucial to Chronic Arthritis Pain and Neuropathic Pain.

**Fig. 1.**
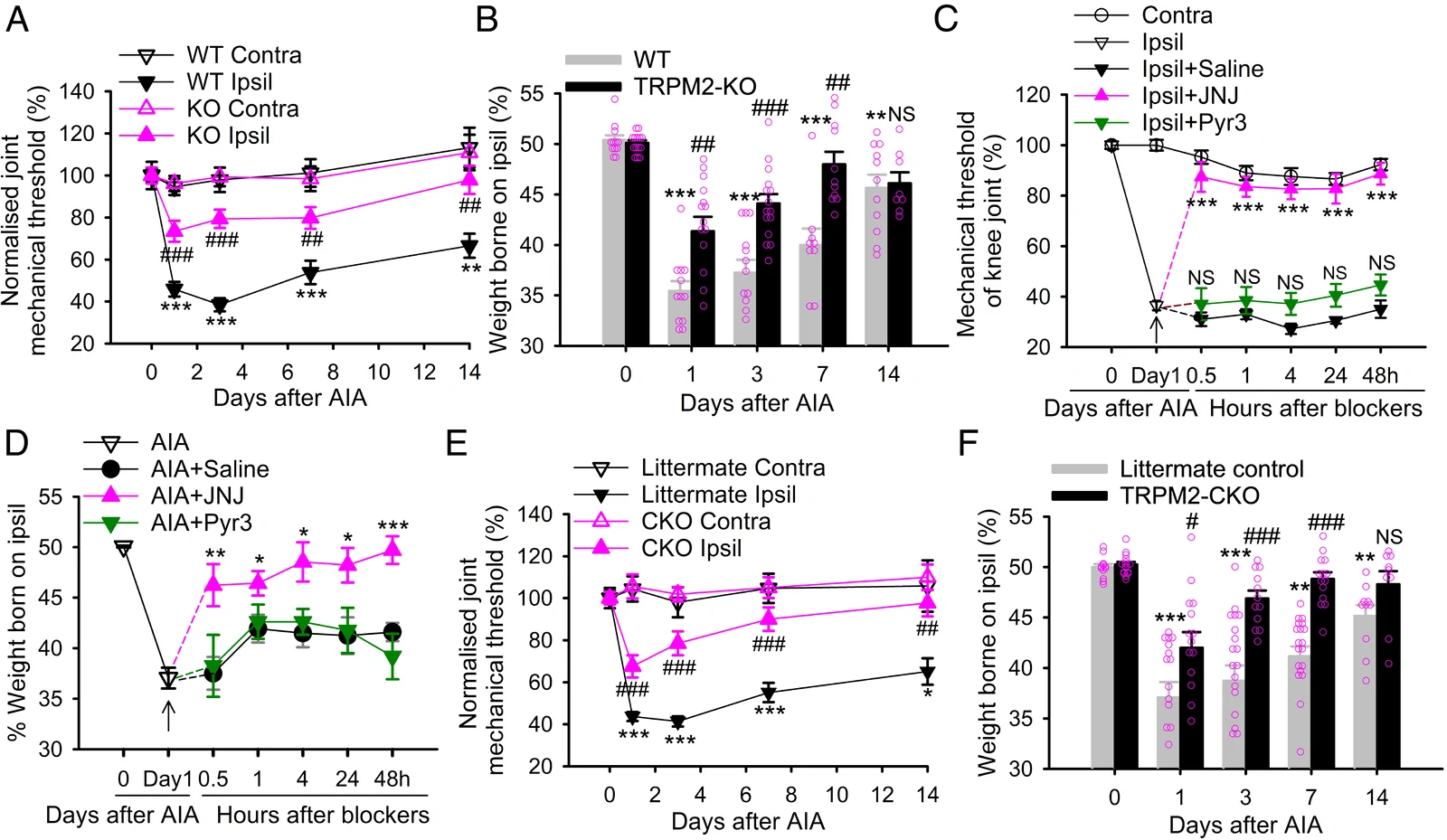
Neuronal TRPM2 channels transduce chronic arthritis pain. (*A*) Mechanical threshold in the contralateral and ipsilateral knee joints in WT and TRPM2-KO mice after AIA induction. n = 6 to 19/group. (*B*) Percentage of body weight borne on the ipsilateral limb in AIA mice. n = 8 to 15 per group. (*C* and *D*) Effects of JNJ-28583113 (2 mM, 10 μL), Pyr3 (2 mM, 10 μL), and saline on knee joint mechanical threshold (*C*) and body weight distribution (*D*) in AIA mice. n = 5 to 19 per group. arrows indicate drug injection (i.art). ***; NS (not significant), compared to saline. (*E* and *F*) Knee joint mechanical threshold (*E*) and body weight distribution on the ipsilateral limb (*F*) in TRPM2-CKO AIA mice. n = 6 to 19.

To determine the role of TRPM2 in chronic arthritis pain, we employed antigen-induced arthritis (AIA), an animal model of rheumatoid arthritis. Mice were preimmunized by methylated BSA (mBSA) followed by intraarticular injection of mBSA into one knee joint (26). Joint mechanical pain rapidly developed one day after injection, peaking at around day 3 and then gradually attenuated, but significant joint pain was still evident after 14 d (Fig. 1*A*). Similar results were obtained with weight bearing tests in which primary joint pain was measured by monitoring body weight distribution difference between the contralateral and ipsilateral limbs of mice (Fig. 1*B*). Compared to wild-type (WT) mice, joint pain was markedly reduced in TRPM2-knockout (KO) mice (Fig. 1 *A* and *B*). We then tested the effect of pharmacological blockade of TRPM2 one day after AIA by injecting the specific TRPM2 antagonist JNJ-28583113 into the knee joint (27). Strikingly, joint pain was entirely reversed, and the analgesic effect persisted for two days (Fig. 1 *C* and *D*), though no appreciable effect of JNJ-28583113 on joint inflammation was detected (*SI Appendix*, Fig. S1*A*), suggesting that persistent TRPM2 activity in joint sensory nerve endings (i.e., neuronal TRPM2) drives arthritis pain.

To specifically investigate the role of neuronal TRPM2 and exclude the indirect effect of immune TRPM2 on chronic pain, we generated TRPM2 conditional knockout (TRPM2-CKO) mice by crossing homozygous floxed TRPM2 mice (TRPM2^fl/fl^) with Advillin^Cre^ mice in which Cre is exclusively expressed in DRG neurons (28). We found that TRPM2 mRNA and protein were expressed in 40.6% (432/1065) and 46.4% (1195/2576) of DRG neurons, respectively (*SI Appendix*, Fig. S1 *B* and *F*), compatible with the expression of functional TRPM2 in 37% of DRG neurons (24). 42.9% (513/1195) of TRPM2^+^ DRG neurons coexpressed TRPV1, and 40.8% (498/1219) and 15.9% (118/744) of TRPM2^+^ DRG neurons coexpressed substance P and IB4, respectively (*SI Appendix*, Fig. S1 *B*–*E*). As expected, TRPM2 expression was ablated in the DRG, spinal cord, and infiltrated neutrophils in the arthritis joints (AIA) from TRPM2-KO mice (*SI Appendix*, Fig. S1 *G* and *H*), demonstrating the specificity of TRPM2 antibody. In contrast, evident TRPM2 expression was observed in the spinal cord and joint neutrophils but lost in the DRG from TRPM2-CKO mice (*SI Appendix*, Fig. S1 *G* and *H*), demonstrating that TRPM2 is selectively ablated from the DRG in TRPM2-CKO mice. Intriguingly, TRPM2-CKO mice reproduced all the pain deficits in TRPM2-KO mice (Fig. 1 *E* and *F*), suggesting that neuronal TRPM2 directly transduces chronic arthritis pain independently of immune TRPM2.

**Fig. 2.**
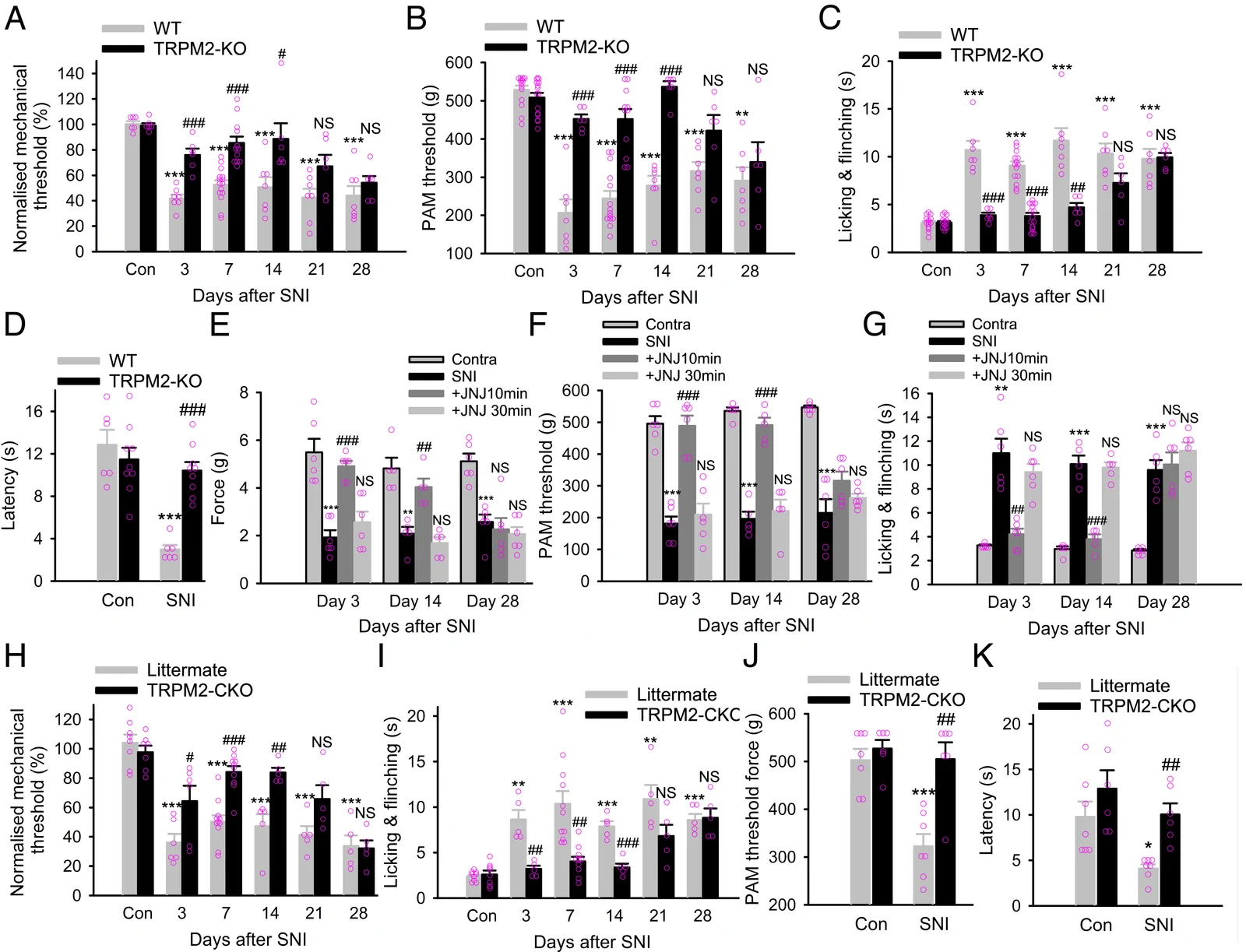
Neuronal TRPM2 channels transduce chronic neuropathic pain. (*A*–*K*) Paw withdrawal threshold force to von Frey probe (*A*, *E*, and *H*) and PAM device (*B*, *F*, and *J*) and licking and flinching time to acetone (*C*, *G*, and *I*) and Paw withdrawal latency to infrared heat (*D* and *K*) in mice at different days (*A*–*C*, and *E*–*I*) or 7 d (*D*, *J*, and *K*) after SNI surgery in the contralateral (Con) and ipsilateral (SNI) paws in WT and TRPM2-KO mice (*A*–*D*) and TRPM2-CKO mice and littermates (*H*–*K*) and in WT mice preinjected (i.pl) with JNJ-28583113 (2 mM) (*E*–*G*). *, **, *** compared to the contralateral paws/joints; #, ##, ### compared to the ipsilateral paws/joints in WT or littermate mice.

We then examined the role of neuronal TRPM2 in neuropathic pain caused by spared nerve injury (SNI), a different type of chronic pain. Prominent mechanical and cold allodynia were developed 3 d after nerve injury and maintained afterward with little attenuation even at day 28 of post-SNI (Fig. 2 *A*–*C*). Heat hyperalgesia was also evident (Fig. 2*D*). Interestingly, pain hypersensitivity was abolished prior to 14 d after nerve injury but resurged at day 28 of postsurgery in TRPM2-KO mice (Fig. 2 *A*–*C*), suggesting that the mechanisms of the early and late stages of neuropathic pain are different and that TRPM2 is critical to the early but not late stage of neuropathic pain. The resurged late stage of neuropathic pain is likely caused by central sensitization. Consistent with this idea, pharmacological blockade of TRPM2 with JNJ-28583113 rapidly reversed the early stage of neuropathic pain but had no effect on the late stage of neuropathic pain at day 28 of postinjury (Fig. 2 *E*–*G*). The pain-reversing effect of JNJ-28583113 lasted for less than 30 min different from the long-lasting analgesic effect on arthritis pain (Figs. 1*C* and 2 *E–G*). All these pain deficits were again recapitulated in TRPM2-CKO mice (Fig. 2 *H*–*K*), further consolidating that neuronal but not immune TRPM2 is a predominant player in chronic pain.

### Effect of TRPM2 on Immune-Inflammation and Neurogenic Inflammation.

**Fig. 3.**
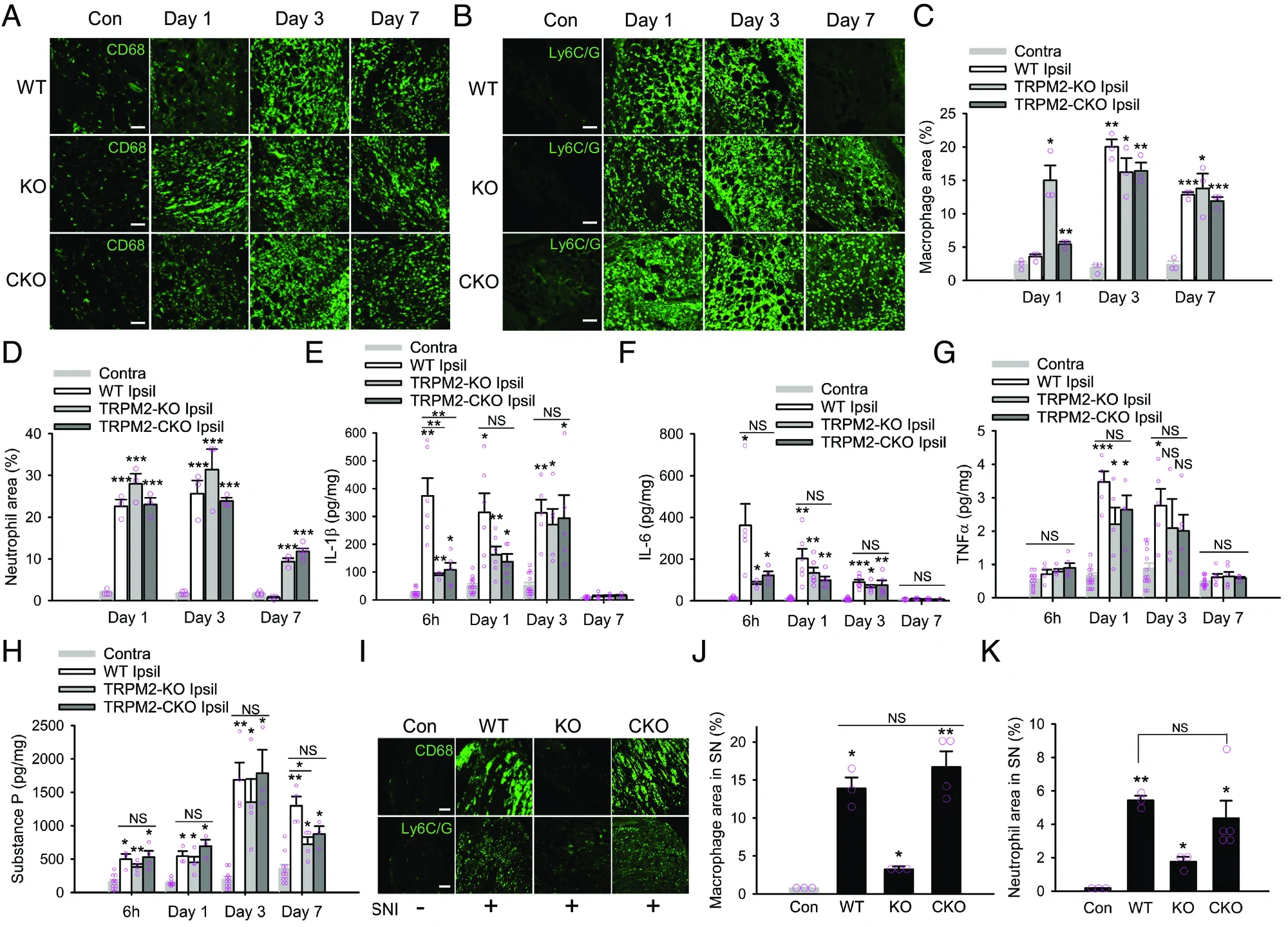
Effect of TRPM2 on tissue inflammation in AIA and SNI models. (*A*) Example images of CD68^+^ macrophages in the synovial tissues of knee joints in different days after AIA. (Scale bar, 50 μm.) (*B*) Representative staining of neutrophils by anti-Ly6C/G in synovial tissues of knee joints from different days of AIA mice. (*C*) Collective results of macrophage-occupied area from similar experiments to those in (*A*). (*D*) Summary of results from experiments similar to those in (*B*). (*E*–*H*) Concentrations of IL-1β (*E*), IL-6 (*F*), TNFα (*G*) and SP (*H*) measured by ELISA in the knee joints of mice after different days of AIA induction. (*I*–*K*) Representative images (I) and summary (*J* and *K*) of CD68^+^ macrophages (*J*) and Ly6C/G^+^ neutrophils (*K*) in the sciatic nerves from SNI mice. (Scale bar, 50 μm.) NS, not significant.

Immune and inflammatory cells are recognized as important players in chronic arthritis pain (29). To determine the effect of TRPM2 on joint inflammation in chronic arthritis, we analyzed infiltration of inflammatory cells using specific markers including CD68 for macrophages, Ly6C/G for neutrophils/monocytes, CD3 for lymphocytes, and toluidine blue staining of mast cells in the knee joints of mice. We found that joint macrophages were markedly increased, peaking around day 3 (Fig. 3*A* and *SI Appendix*, Fig. S2*A*), consistent with a critical role of macrophages in inflammatory arthritis (29). However, deletion of TRPM2 did not prevent macrophage recruitment and even enhanced the recruitment at day one of post-AIA (Fig. 3 *A* and *C*). Macrophage recruitment in TRPM2-CKO mice was similar to that in WT mice (Fig. 3 *A* and *C*), suggesting that pain deficits in TRPM2 mutant mice are not likely mediated by macrophages. Neutrophils were also robustly increased in the knee joints of WT mice at day one and day 3 but resolved at day 7 of post-AIA (Fig. 3*B* and *SI Appendix*, Fig. S2*B*). Equivalent neutrophil recruitment was also found in TRPM2-KO and -CKO mice and remained prominent at day 7 (Fig. 3 *B* and *D*). The antibodies used for staining CD68 and Ly6C/G are specific, because these antibodies revealed a single protein band and furthermore no evident background staining was observed in the absence of primary antibodies (*SI Appendix*, Fig. S2*C*). Similarly, joint lymphocytes were markedly increased in WT and TRPM2-CKO mice but not in TRPM2-KO mice (*SI Appendix*, Fig. S2 *D* and *E*), implying that immune but not neuronal TRPM2 is crucial to lymphocytes infiltration. We further analyzed joint mast cells and DRG macrophages, but no difference was observed between all the mice genotypes (*SI Appendix*, Fig. S2 *F*–*I*). Furthermore, joint pathology including synovial hyperplasia and cartilage damage was indistinguishable between WT and TRPM2 mutant mice (*SI Appendix*, Fig. S3 *A*–*D*). Collectively, our data suggest that neuronal TRPM2 is involved in neither joint inflammatory cell infiltration nor joint damage, though immune TRPM2 is critical to lymphocyte infiltration in arthritic joints. Therefore, chronic arthritis pain transduced by neuronal TRPM2 is unlikely mediated by these inflammatory cells.

We next analyzed major cytokines and neuropeptides critical to joint inflammation and arthritis pain in the knee joints using ELISA assay (30, 31). IL-1β and IL-6 were dramatically increased 6 h after AIA but TNFα was not increased until one day after AIA in the ipsilateral joints of WT mice (Fig. 3 *E*–*G*). However, all these cytokines declined to the basal level 7 d after AIA, suggesting that these cytokines play a role in the early but not chronic stage of arthritis pain. However, no significant difference was found in the production of these cytokines in either TRPM2-KO or -CKO mice except IL-1β at 6 h (Fig. 3 *E*–*G*). These results suggest that these cytokines are not crucial to TRPM2-mediated arthritis pain, though IL-1β may contribute to the early stage of arthritis pain.

We further examined Substance P (SP) and CGRP, two primary neuropeptides critical to neuroimmune crosstalk implicated in rheumatoid arthritis (32–34). SP was markedly increased at all the tested time points, peaking at day 3 but remaining high at day 7 of post-AIA (Fig. 3*H*), supporting an important role of SP in both acute and chronic stages of arthritis. However, no significant differences were observed between WT, TRPM2-KO, and -CKO mice, except that KO mice exhibited a mild reduction at day 7 (Fig. 3*H*). Surprisingly, there was no change in CGRP between the contralateral and ipsilateral joints of AIA across all mice genotypes (*SI Appendix*, Fig. S3*E*). Thus, TRPM2-mediated arthritis pain is unlikely due to neurogenic inflammation.

To determine the contribution of immune-inflammation to neuropathic pain, similar approaches were used to detect macrophages and neutrophils in the DRG and sciatic nerves 7 d after nerve injury. Consistent with others (35–38), DRG macrophages were significantly increased but indistinguishable between WT, TRPM2-KO, and -CKO mice (*SI Appendix*, Fig. S4 *A* and *B*). Macrophages were also dramatically increased at the injury site of sciatic nerves in both WT and TRPM2-CKO mice but absent in TRPM2-KO mice (Fig. 3 *I* and *J*). Similarly, neutrophil infiltration was equivalently increased in WT and TRPM2-CKO mice, but not in TRPM2-KO mice (Fig. 3 *I* and *K*). These results support that neuronal TRPM2 does not affect the infiltration and recruitment of inflammatory cells at either injury site or DRG during nerve injury but in contrast, immune TRPM2 is indispensable for macrophage and neutrophil infiltration at nerve injury site though it is not required for the recruitment of DRG macrophages. Given that DRG macrophages but not nerve macrophages are critical to the initiation and maintenance of nerve injury-induced neuropathic pain (35, 38), our data support that neuronal TRPM2 directly transduces neuropathic pain independent of immune TRPM2 nor by indirectly affecting inflammatory cells.

Taken together, our results demonstrate that neuronal TRPM2 directly transduces both chronic arthritis pain and neuropathic pain with little involvement of immune TRPM2 nor by indirectly affecting immune-inflammation or neurogenic inflammation.

### Neuronal TRPM2 Transduces Pain Signaling Evoked by PGE2.

We then aimed to understand the common mechanisms of chronic arthritis pain and neuropathic pain carried by neuronal TRPM2. ADPR is an endogenous metabolite agonist for TRPM2 channels and is increased by oxidative stress (16, 17). Given that chronic pain is accompanied by oxidative stress, it seems plausible that increased ADPR activates TRPM2 promoting chronic pain. However, neither DRG nor sciatic nerves exhibited increased ADPR in AIA and SNI models (*SI Appendix*, Fig. S5 *A*–*D*). Therefore, TRPM2-mediated chronic pain is unlikely caused by ADPR.

**Fig. 4.**
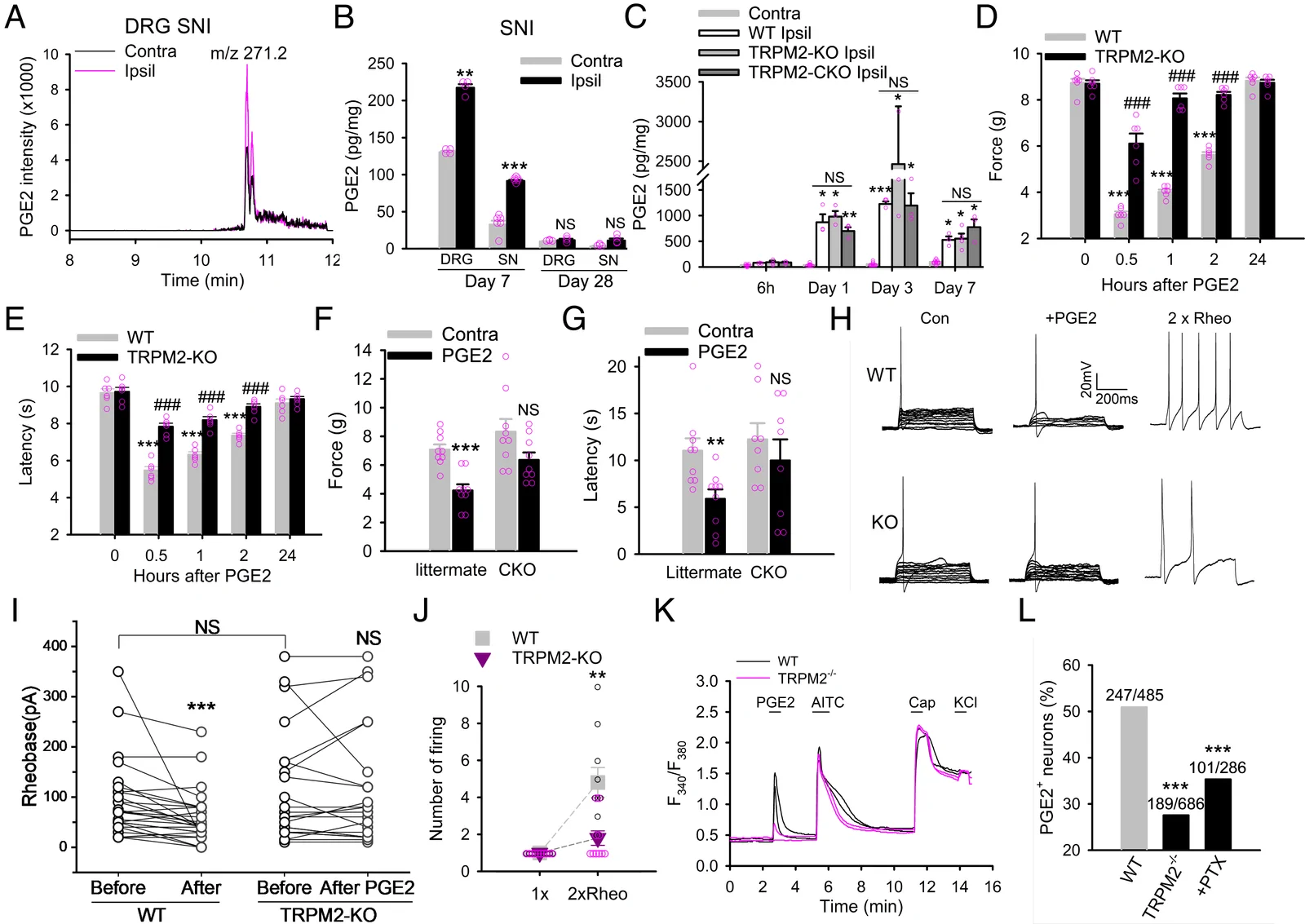
Neuronal TRPM2 mediates PGE2-induced acute pain. (*A*) LC–MS analysis of PGE2 in the contralateral and ipsilateral DRG from SNI mice. (*B*) Summary of PGE2 in DRG and sciatic nerves (SN) from experiments similar to those in (*A*). n = 4 to 6 per group. (*C*) PGE2 concentration in the knee joints from mice after different days of AIA induction measured with ELISA. (*D*–*G*) Threshold mechanical force (*D* and *F*) and withdrawal latency to infrared heat (*E* and *G*) in the paws of WT and TRPM2-KO (*D* and *E*), TRPM2-CKO, and littermate mice (*F* and *G*, 0.5 h) after intraplantar injection of PGE2 (10 μg/10 μL). (*H*) Example firing of DRG neurons from WT and TRPM2-KO mice evoked by depolarization current steps (*Left* and middle panels) and 2-times rheobase current (*Right* panel) before and after PGE2 treatment (1 μM). (*I*) Summary of results from experiments similar to those in (*H*). (*J*) Summary of number of firing induced by 1x and 2x rheobase current. (*K*) Example Ca^2+^ responses in DRG neurons induced by PGE2 (1 μM), AITC (100 μM), capsaicin (1 μM), and KCl (50 mM). (*L*) Summary of PGE2-responding DRG neurons from WT and TRPM2^–/–^ mice and WT DRG neurons treated with pertussis toxin (PTX, 1 μg/mL 12 h).

PGE2 is an important player in the pathogenesis of both rheumatoid arthritis pain and neuropathic pain (39–42). Indeed, we found that PGE2 was markedly increased in both DRG and sciatic nerve 7 d after nerve injury but declined to the basal level at day 28 of postinjury (Fig. 4 *A* and *B*). In the AIA model, PGE2 in the knee joints was not altered 6 h after AIA but then robustly increased after one day, lasting for over 7 d (Fig. 4*C*), supporting a key role for PGE2 in sustaining chronic arthritis pain. Comparable increase in joint PGE2 was also observed in TRPM2-KO and -CKO mice and was not significantly different compared to that in WT mice (Fig. 4*C*). PGE2 was also significantly increased in the ipsilateral DRG one day after AIA induction but then diminished to an insignificant level after 3 d compared to that in the contralateral DRG (*SI Appendix*, Fig. S5*E*). No changes in PGE2 in sciatic nerves were observed in the AIA model (*SI Appendix*, Fig. S5*F*). These results suggest that local inflamed joint tissues are a primary source of PGE2 responsible for prolonged arthritis pain and that TRPM2 is not involved in PGE2 production.

To determine whether TRPM2 transduces acute pain induced by PGE2, the hind paws of mice were injected with PGE2. Mechanical and heat hypersensitivity were rapidly developed 0.5 h after PGE2 injection and then gradually declined to the basal level after 24 h (Fig. 4 *D* and *E*). However, heightened pain was prevented in TRPM2-KO mice (Fig. 4 *D* and *E*), and the pain deficit was recapitulated in TRPM2-CKO mice (Fig. 4 *F* and *G*), demonstrating that PGE2-induced acute pain is mainly mediated by neuronal TRPM2. At the cellular level, PGE2 enhanced the excitability of sensory DRG neurons, as indicated by reduced rheobase and increased firing frequency caused by PGE2 (Fig. 4 *H*–*J*). Neuronal hyperexcitability was, however, abolished in TRPM2-lacking DRG neurons (Fig. 4 *H*–*J*). Apart from nociceptor sensitization, PGE2 also triggers spontaneous nociceptor firing, which has been recently shown to be essential to drive mechanical allodynia (43). However, neither TRPV1 nor TRPA1 mediate spontaneous firing evoked by PGE2 (43, 44). We also found that PGE2 induced spontaneous firing in 16.2% (6/37) of WT DRG neurons (*SI Appendix*, Fig. S6 *A* and *B*). Interestingly, spontaneous firing evoked by PGE2 was rapidly inhibited by blocking TRPM2 with JNJ-28583113, and also significantly reduced in TRPM2-lacking DRG neurons (*SI Appendix*, Fig. S6 *B*–*D*), suggesting that TRPM2 mediates spontaneous firing caused by PGE2. Consistently, the percentage of DRG neurons responding to PGE2 was also markedly reduced in TRPM2-deficient mice (Fig. 4 *K* and *L*).

These results support that neuronal TRPM2 directly transduces PGE2-induced acute pain through enhancing the excitability and direct activation of sensory DRG neurons.

### Neuronal TRPM2 Transduces Acute Pain Elicited by IgG-IC.

**Fig. 5.**
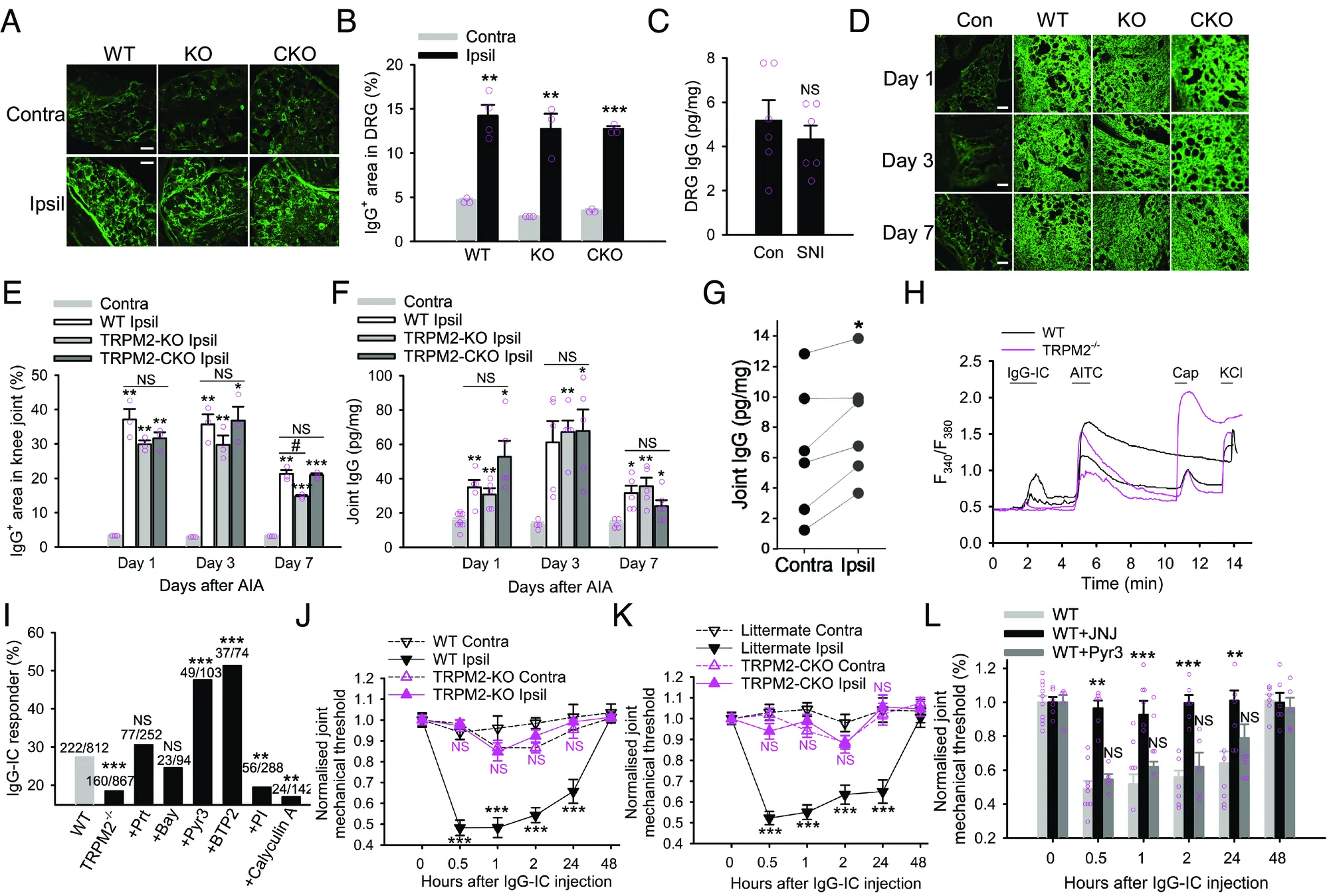
Neuronal TRPM2 transduces acute pain evoked by IgG-IC. (*A*) IgG immunoreactivity in the contralateral and ipsilateral lumbar DRG 7 d after SNI. (Scale bar, 50 μm.) (*B*) Summary of IgG^+^ area in the lumbar DRG from experiments similar to those in (*A*). (*C*) IgG determined by ELISA in lumber DRG 28 d after SNI. (*D*) Representative staining of IgG immunoreactivity in the knee joint of WT, TRPM2-KO, and TRPM2-CKO mice after AIA induction. (Scale bar, 50 μm.) (*E*) Summary of IgG^+^ staining area from experiments similar to those in (*D*). (*F*) Summary of IgG concentration measured by ELISA in the knee joints from WT, TRPM2-KO, and TRPM2-CKO mice after AIA induction. n = 4 to 9 per group. (*G*) IgG quantification from the contralateral and ipsilateral knee joints of WT mice 28 d after AIA. (*H*) Ca^2+^ responses in WT and TRPM2^–/–^ DRG neurons evoked by IgG-IC (1 μg/mL), AITC (100 μM), capsaicin (1 μM), and KCl (50 mM). (*I*) Summary of IgG-IC-sensitive DRG neurons from similar experiments to those in (H) in the presence of different inhibitors, Prt062607 (Prt, 0.3 μM), Bay-61-3606 (Bay, 10 μM), Pyr3 (10 μM), BTP2 (10 μM), phosphatase inhibitor cocktail (1:100), and Calyculin A (0.2 μM). (*J* and *K*) Mechanical threshold of the contralateral and ipsilateral knee joints of WT/TRPM2-KO mice (*J*) and TRPM2-CKO mice/littermate (*K*) after intra-articular injection of IgG-IC (2 μg). (*L*) Effect of JNJ-28583113 (2 mM) and Pyr3 (2 mM) on mechanical threshold in the knee joints of WT mice after IgG-IC injection (i.art).

Apart from PGE2, recent evidence suggests that IgG-IC is a cause of both arthritis pain and neuropathic pain (3–6, 8, 9). Consistently, we found that nerve injury significantly increased IgG immunoreactivity in the ipsilateral lumbar DRG compared to that in the contralateral DRG in WT mice 7 d after nerve injury (Fig. 5*A* and *SI Appendix*, Fig. S7*A*). Equivalent increase in IgG immunoreactivity was also evident in TRPM2-KO and CKO mice (Fig. 5 *A* and *B*). However, there was no significant difference in lumber DRG IgG 28 d after nerve injury (Fig. 5*C*), in line with insignificant difference in DRG PGE2 at the late stage of SNI (Fig. 4*B*), all of which may determine the critical role of TRPM2 in the early but not chronic stage of neuropathic pain (Fig. 2). As expected, IgG accumulation was also drastically increased in the knee joints of mice one day after AIA but then decreased at day 7 (Fig. 5 *D* and *F* and *SI Appendix*, Fig. S7*B*). However, IgG was still significantly increased in the ipsilateral joints even at day 28 of post-AIA (Fig. 5*G*). Accumulated IgG-IC was not affected by deleting TRPM2 (Fig. 5 *D* and *F*). In contrast to the knee joints, IgG immunoreactivity in the DRG was not altered in AIA mice (*SI Appendix*, Fig. S7 *C* and *D*), suggesting that joint IgG is a primary cause of arthritis pain.

It has been shown that IgG-IC directly excites DRG neurons through activating Syk and TRPC3 channels (45, 46). We also found that a brief application of IgG-IC activated 27.3% DRG neurons, 98.7% of which expressed TRPA1 and/or TRPV1 (Fig. 5 *H* and *I*). However, we found that IgG-IC-responding neurons were not affected by two different Syk inhibitors Prt062607 and Bay-61-3606 (Fig. 5*I*). Surprisingly, two specific TRPC3 channel inhibitors Pyr3 and BTP2 even enhanced Ca^2+^ responses evoked by IgG-IC (Fig. 5*I*). Pyr3 and BTP2 also elicited basal Ca^2+^ increases in 41.4% (46/111) and 47.2% (52/110) of DRG neurons, respectively (*SI Appendix*, Fig. S8 *A* and *B*), which is likely caused by enhanced basal activity of IP3R in the ER due to constitutive inhibition of IP3R by TRPC3 (47). These results challenge the suggestion of TRPC3 as a downstream channel responsible for IgG-IC-induced neuronal responses (48). Nevertheless, IgG-IC responders were significantly reduced in TRPM2-lacking DRG neurons (Fig. 5 *H* and *I*). Similar inhibitory effect was also observed by inhibiting PP1/PP2A serine/threonine phosphatases (Fig. 5*I*), suggesting that neuronal excitation evoked by IgG-IC is mediated by neuronal TRPM2 and serine/threonine phosphatases (see below) but not by Syk nor TRPC3.

To determine whether neuronal TRPM2 transduces IgG-IC-induced acute pain in vivo, we injected IgG-IC into the knee joints of mice. Joint mechanical pain was rapidly developed 0.5 h after injection and lasted for 2 d, though no significant joint inflammation was observed (Fig. 5*J* and *SI Appendix*, Fig. S7*E*), consistent with the notion that IgG-IC directly excites joint sensory nerve endings triggering joint pain independently of inflammation (5, 6, 8, 46). However, joint mechanical pain was all abolished in both TRPM2-KO and TRPM2-CKO mice (Fig. 5 *J* and *K*). Moreover, pharmacological blockade of TRPM2 with JNJ-28583113 also entirely reversed acute joint pain induced by IgG-IC, whereas TRPC3 inhibitor (Pyr3) had no effect (Fig. 5*L*). Consistently, chronic arthritis pain was not affected by Pyr3 but eliminated by JNJ-28583113 (Fig. 1 *C* and *D*). These data strongly demonstrate that neuronal TRPM2 is a predominant channel transducing both acute joint pain and chronic arthritis pain caused by IgG-IC, whereas TRPC3 is dispensable, consistent with a recent report that TRPC3 channels in sensory DRG neurons are not involved in pain transduction (49).

Altogether, these experiments demonstrate that neuronal TRPM2 channels directly transduce IgG-IC evoked pain.

### PGE2 and IgG-IC Activate TRPM2 Channels Independently of ADPR.

**Fig. 6.**
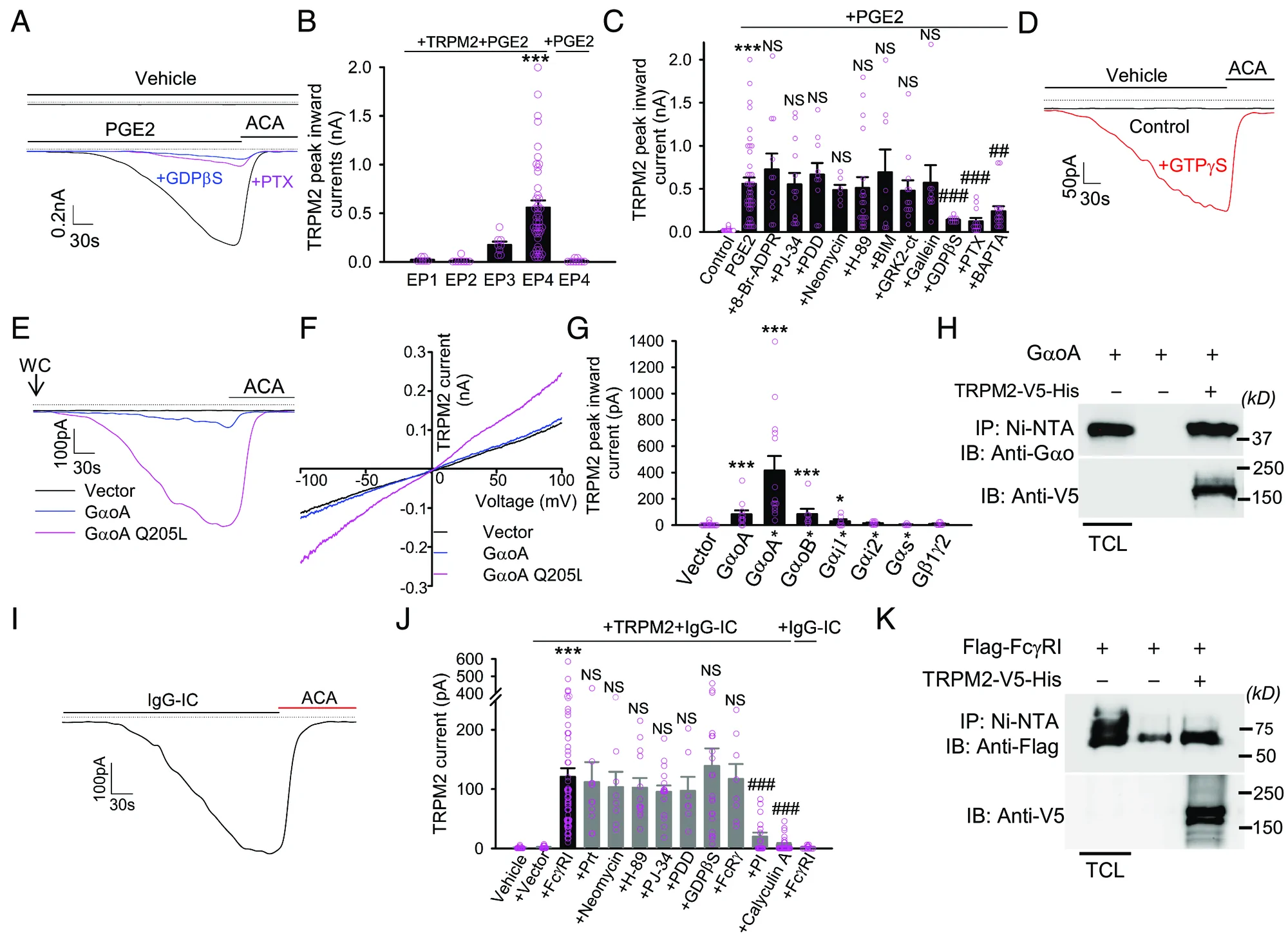
TRPM2 is activated by IgG-IC and PGE2 through noncanonical mechanisms. (*A*) Example current traces in HEK293 cells expressing TRPM2 and EP4 receptor evoked by vehicle and PGE2 (1 μM) or after treatment with GDPβS (0.5 mM) and pertussis toxin (PTX, 1 μg/mL 12 h). Current was blocked by *N*-(p-amylcinnamoyl)anthranilic acid (ACA, 20 μM). (*B*) Summary of TRPM2 currents mediated by EP1-4 from similar experiments to those in (*A*) or in cells expressing EP4 but without TRPM2 (last bar). (*C*) Collective results from experiments similar to those in (*A*) in cells pretreated with 8-Br-ADPR (300 μM), PJ-34 (20 μM), PDD00017293 (10 μM), neomycin (1 mM), H-89 (10 μM), BIM (1 μM), GRK2-ct, gallein (100 μM), GDPβS and PTX. ***, compared to control; NS, ### compared to the 2nd bar. (*D*) Example currents recorded from HEK293 cells expressing TRPM2 perfused with vehicle with pipette solution containing GTPγS (100 μM) or control solution. (*E*) Example currents in HEK293 cells expressing vector, GαoA and GαOA Q205L induced shortly after breaking into whole-cell (WC). Currents blocked by ACA (20 μM). (*F*) Average current-voltage curve in HEK293 cells expressing TRPM2 together with vector, GαoA or GαoA Q205L from similar experiments to those in (*E*). n = 8 to 12. (*G*) Summary of TRPM2 peak inward currents in HEK293 cells expressing TRPM2 together with vector or different G protein subunits similar to those in (*E*). GαoA* denotes constitutively activated GαoA Q205L mutant, which applies to all other Gα mutants. (*H*) TRPM2 binds to GαoA in a Ni-NTA pull down assay. Lane 1 is total cell lysate (TCL). n = 3. (*I*) Inward current evoked by IgG-IC (1 μg/mL) from HEK293 cells expressing TRPM2 and FcγRI. Currents blocked by ACA (20 μM). (*J*) Summary of results from experiments similar to those in (*I*) but treated with vehicle or with Prt062607 (2 μM), neomycin (1 mM), H-89 (10 μM), PJ-34 (20 μM), PDD00017293 (10 μM), GDPβS (0.5 mM), coexpression with FcRγ, phosphatase inhibitor cocktails (1:100) and calyculin A (200 nM), or in cells cotransfected with vector and TRPM2 (bar 2) or in cells expressing FcγRI alone without TRPM2 (last bar). ***, compared to control; NS, ###, compared to the 2nd bar. (*K*) TRPM2 binds to Flag-FcγRI in a nickel beads pull down assay. n = 3.

Our behavioral and cellular studies suggest that PGE2 and IgG-IC activate TRPM2 channels mediating acute and chronic pain. To test this idea, we used whole-cell patch clamping to record TRPM2 currents activated by PGE2 in HEK293 cells expressing PGE2 receptors. We found that PGE2 but not vehicle, evoked a large TRPM2-dependent inward current in cells coexpressing EP4 receptor (Fig. 6*A*). Furthermore, no currents were elicited by PGE2 in the absence of TRPM2 coexpression (Fig. 6*B*). TRPM2 currents were also elicited by activation of EP3 receptors albeit to a lesser degree, but neither EP1 nor EP2 had any effects (Fig. 6*B*). TRPM2 currents are unlikely solely caused by Ca^2+^, a known activator of TRPM2 channels (13, 50–52), because [Ca^2+^]_i_ was buffered in our recordings (bath solution, 1 mM Ca^2+^; pipette solution, 2 mM EGTA) below 150 nM, which is insufficient to activate TRPM2 (51). Significant TRPM2 currents were still evoked by PGE2 even when [Ca^2+^]_i_ was buffered with the rapid Ca^2+^ chelator BAPTA (2 mM), though TRPM2 activation was reduced (Fig. 6*C*), supporting that PGE2 is sufficient to open TRPM2 channels through EP4 receptor independently of Ca^2+^, though Ca^2+^ influx through the opened channel pore likely further amplifies TRPM2 activation forming a positive feedback (51).

We then investigated the signaling mechanisms of TRPM2 activation by PGE2. TRPM2 activation was not affected by 8-Br-ADPR, an ADPR antagonist (53), nor by PJ-34 and PDD00017273 (Fig. 6*C*), inhibitors for PARP-1 and PARG, respectively, critical to ADPR production. ADPR is thus not involved in TRPM2 activation, consistent with the absent effect of chronic arthritis pain and neuropathic pain on ADPR production (*SI Appendix*, Fig. S5 *A*–*D*). No effects were observed, either, by inhibiting phospholipase C with neomycin, inhibiting PKA and PKC with H-89 and Bisindolylmaleimide, respectively, nor by inhibiting/chelating Gβγ with gallein and Mas-GRK2-ct (Fig. 6*C*). However, it was inhibited by inactivating G proteins through dialyzing cells with nonhydrolyzable GDP analogue GDP-β-S or by inactivating Gαi/o with pertussis toxin (PTX) (Fig. 6*C*). Conversely, activation of endogenous G proteins by intracellular application of GTPγS was sufficient to activate TRPM2 (Fig. 6*D*). These results suggest that PGE2 activates TRPM2 through Gαi/o. The conclusion is further supported by our finding that TRPM2 is activated by Gαi/o-coupled EP3 and EP4 receptors but not by EP1 and EP2 receptors coupled to Gαq and Gαs, respectively (Fig. 6*B*). Collectively, these results demonstrate that Gαi/o signaling is a TRPM2 activator independently of Ca^2+^, ADPR, and other conventional downstream messengers.

In experiments aiming to identify the Gαi/o subunits responsible for activating TRPM2, we found that GαoA but not vector cotransfection is sufficient to trigger TRPM2-dependent inward currents shortly after breaking cells into whole-cell configuration (Fig. 6*E*). Constitutively activated GαoA Q205L mutant provoked even more prominent TRPM2 currents and enhanced voltage-dependent gating of TRPM2 (Fig. 6 *E* and *F*). Appreciable TRPM2 activation was also triggered by activated GαoB and Gαi1, but other G protein subunits including Gαi2, Gαs and Gβ1γ2 had negligible effect (Fig. 6*G*). Furthermore, GαoA were found to bind TRPM2 in a nickel beads pull down assay (Fig. 6*H*), suggesting that GαoA directly activates TRPM2 through physical interaction. In support of this idea, Gαo proteins were abundantly expressed in DRG neurons (54). To ascertain whether Gαi/o proteins are also responsible for TRPM2 activation in native DRG neurons, pertussis toxin (PTX) was used to inactivate Gαi/o proteins by treating the cells overnight. We found that PTX treatment markedly inhibited Ca^2+^ responses in DRG neurons induced by PGE2 (Fig. 4*L*). Thus, our results suggest that GαoA is a TRPM2 activator mediating neuronal excitation caused by PGE2.

We next examined whether IgG-IC activates TRPM2 through FcγRI, known to mediate chronic arthritis pain and neuropathic pain caused by autoantibodies (3, 5, 6, 8, 9). Interestingly, we found that IgG-IC perfusion also activated TRPM2 currents in HEK293 cells expressing FcγRI and TRPM2 but not in cells expressing TRPM2 or FcγRI alone (Fig. 6 *I* and *J*), suggesting that IgG-IC activates TRPM2 depending on FcγRI. Of note, the activation was triggered even in the absence of γ chain (Fig. 6*I*), essential to transduce FcγRI signaling initiated by FcγRI crosslinking (7). Furthermore, coexpression with γ chain (FcRγ) did not alter TRPM2 activation by IgG-IC (Fig. 6*J*), suggesting signaling-independent activation of TRPM2 by FcγRI. Consistent with this idea, TRPM2 currents induced by IgG-IC were neither inhibited by Syk2 inhibitor nor by inhibitors for PLC/PKA/PARP-1/PARG nor by inactivating G protein with GDP-β-S (Fig. 6*J*). These data are also supported by our finding of absent roles of Syk inhibitors in [Ca^2+^]_i_ responses in DRG neurons induced by IgG-IC (Fig. 5*F*). Overall, these results suggest that FcγRI activates TRPM2 independent of canonical signaling messengers or G proteins. IgG-IC-induced FcγRI crosslinking may directly activate TRPM2 channels. In support of this idea, TRPM2 bound to FcγRI (Fig. 6*K*), suggesting that TRPM2 and FcγRI form a receptor-channel complex. FcγRI crosslinking requires receptor dephosphorylation by PP1/PP2A serine/threonine phosphatases (55, 56). Interestingly, we found that inhibiting PP1/PP2A with phosphatase inhibitor cocktails and Calyculin A both prevented IgG-IC-induced TRPM2 currents (Fig. 6*J*), supporting that FcγRI crosslinking is sufficient to activate TRPM2. Importantly, both serine/threonine phosphatase inhibitors also potently inhibited Ca^2+^ responses in DRG neurons activated by IgG-IC to a similar extent seen in TRPM2-deficient DRG neurons (Fig. 5*F*), suggesting that FcγRI crosslinking also directly activates TRPM2 channels in native DRG neurons.

Collectively, PGE2 and IgG-IC activate TRPM2 through distinct mechanisms independently of conventional signaling messengers.

## Discussion

Chronic pain is driven by maladaptive neuroimmune responses. As TRPM2 channels are abundantly expressed in immune cells, it has been suggested that TRPM2 contributes to chronic pain by indirectly regulating immune responses through immune TRPM2 (22, 23). Contrary to this view, we found that TRPM2 in sensory neurons (i.e., neuronal TRPM2) directly transduces acute and chronic pain with little involvement of immune TRPM2. Neuronal TRPM2 thus functions as a direct pain transducer. However, neuronal TRPM2 was also proposed as a sensor for innocuous warm temperatures (24). This raised the question of how neuronal TRPM2 transduces both innocuous and noxious signals. Presumably, there exist two distinct subpopulations of TRPM2^+^ DRG neurons transducing pain and temperature, respectively. In support of this possibility, only 3.5% of warmth-sensing DRG neurons are mediated by TRPM2 (24). The vast majority of remaining TRPM2^+^ DRG neurons may be responsible for pain transduction. More research is required to understand this functional dilemma.

Our research revealed neuronal TRPM2 as a common mechanism transducing two different types of chronic pain: rheumatoid arthritis pain and neuropathic pain. Notably, TRPM2 is mainly involved in the early (14 d of postinjury) but not chronic stage (28 d of postinjury) of neuropathic pain. Consistently, PGE2 and IgG in lumbar DRG critical to TRPM2 activation were markedly increased in the early stage of SNI but dropped to the basal level at the late stage of neuropathic pain, suggesting that PGE2 and IgG-IC are the two main agents causing TRPM2 activation mediating the early stage of neuropathic pain. We further uncovered the acting mechanisms of neuronal TRPM2 and found that TRPM2 acts as a convergent pain signaling effector by coupling to FcγRI and EP4, two receptors for autoantibodies and PGE2, respectively, both of which are critical players in rheumatoid arthritis pain, neuropathic pain, and many other types of chronic pain (3, 5, 6, 8, 39, 41, 57). These two receptor-channel coupling mechanisms may constitute a common output machinery underlying the prominent effect of neuronal TRPM2 in chronic pain.

The discovery of functional coupling TRPM2 to both antibody and PGE2 signaling also resolved two important yet unanswered questions: First, autoantibodies generated by immune cells have recently emerged as central mediators of neuroimmune crosstalk and common mechanisms of several different types of chronic pain such as rheumatoid arthritis pain, neuropathic pain, fibromyalgia, and low back pain (3, 6, 8, 9, 57–59). It has been shown that autoantibodies drive rheumatoid arthritis and neuropathic pain through FcγRI in DRG neurons (3–6, 9). But a major unanswered question is how autoantibodies directly excite sensory DRG neurons causing pain. Second, apart from autoantibodies, PGE2 is also known to be central to inflammatory pain, chronic arthritis pain, neuropathic pain, and other pain disorders by sensitizing nociceptive channels (39, 41, 42, 60–62). Notably, apart from nociceptor sensitization, PGE2 also induces spontaneous nociceptor firing, which has recently been shown to be essential to drive mechanical allodynia (43). However, the mechanisms underlying spontaneous nociceptor firing evoked by PGE2 are unknown. Neither TRPV1 nor TRPA1 channels mediate this process (43, 44, 61). Our research demonstrated that activation of TRPM2 channels is a primary channel mechanism mediating the excitatory effect of both antibody and PGE2 signaling; first, acute pain elicited by IgG-IC and PGE2 was abolished by either deleting or blocking neuronal TRPM2; second, the same interventions prevented chronic arthritis pain and neuropathic pain; third, Ca^2+^ responses induced by IgG-IC and PGE2 in DRG neurons were reduced in the absence of TRPM2; last, IgG-IC and PGE2 are sufficient to open TRPM2 channels through FcγRI and EP4, respectively. Surprisingly, PGE2 and IgG-IC activate TRPM2 independently of ADPR, Ca^2+^, and other classical downstream signaling messengers. Instead, activated GαoA protein by PGE2 and FcγRI crosslinking by IgG-IC were found to be responsible for TRPM2 activation. These noncanonical TRPM2 activation and coupling mechanisms will impact our understanding of the diverse physiology and diseases mediated by TRPM2 channels and autoantibodies beyond the sensory biology and pain fields such as autoimmune diseases, neuroimmune interaction, neuroinflammation, thermoregulation, insulin secretion, stroke, aging, and neurodegenerative disease (63).

It should be noted that TRPM2 current recordings were performed when [Ca^2+^]_i_ was buffered to prevent the interference of Ca^2+^-dependent activation of TRPM2 (Fig. 6 *A*, *E*, and *I*). However, under physiological Ca^2+^ condition, activation of PGE2 and FcγRI receptors may also initiate parallel downstream signaling leading to increased [Ca^2+^]_i_, which then further boost direct activation of TRPM2 caused by G proteins and FcγRI crosslinking, respectively.

Despite a prominent role of neuronal TRPM2 in transducing chronic arthritis pain, TRPM2 did not affect SP and CGRP release, in contrast to other nociceptive channels such as TRPV1 and TRPA1 critical to neuropeptide release and neurogenic inflammation (64, 65). Nevertheless, SP was dramatically increased in AIA mice and persisted longer (>7 d) than other cytokines, suggesting a more important role for SP in the initiation and maintenance of chronic arthritis pain. Consistent with this idea, SP was found to stimulate synoviocyte proliferation and PGE2 release (32). PGE2 was also stimulated by IL-1β, IL-6, TNFα and IgG-IC (66–69), all of which were critical to chronic pain. Delayed increase in PGE2 in arthritic joints further supports stimulation of PGE2 by these proinflammatory agents (Fig. 4*C*). Increased PGE2 was then coupled to TRPM2 forming a convergent pain signaling pathway which may underlie the pivotal role of neuronal TRPM2 in pain transduction.

In contrast to SP, CGRP was not increased in the joints of AIA mice, suggesting that CGRP does not contribute to enhanced arthritis pain in the AIA model, contrasting with others’ report of involvement of CGRP in rheumatoid arthritis (33, 34). Different animal models may underlie the difference. Overall, chronic arthritis pain transduced by TRPM2 is unlikely mediated by neuropeptide-mediated neurogenic inflammation.

Role of immune TRPM2 in immune-inflammation has been widely investigated but yielding variable effects. It was initially found that immune TRPM2 expressed in immune cells are proinflammatory by promoting release of cytokines and chemokines from monocytes and macrophages exacerbating neutrophil infiltration (18–20, 70). However, others found that TRPM2 inhibited cytokines (IL-1β, IL-6 and TNFα) and chemokine (CXCL2) production and inflammatory cell infiltration through inhibiting NADPH oxidase and ROS production, exerting an anti-inflammatory effect (71–73). We also found similar opposing effects of TRPM2 depending on tissues. In the AIA model, immune TRPM2 promoted recruitment of lymphocytes in the knee joints, but neither macrophage nor neutrophil recruitment in the inflamed joints required TRPM2 (Fig. 3 *A*–*D* and *SI Appendix*, Fig. S2 *A* and *B*). TRPM2 even inhibited macrophage and neutrophil recruitment (Fig. 3 *A* and *B*). This inhibitory effect is unlikely mediated by CGRP, because CGRP release was not influenced by TRPM2, even though CGRP was shown to inhibit macrophage and neutrophil recruitment (74, 75). Apart from inflammatory cells in the joints, DRG macrophages were reported to be increased in the K/BxN serum-transfer rheumatoid arthritis model and proposed as a mechanism of arthritis pain (33, 76). However, we did not find difference in macrophages between the contralateral and ipsilateral DRG in the AIA model (*SI Appendix*, Fig. S2 *E* and *F*), consistent with a previous research showing no difference in macrophages between the contralateral and ipsilateral DRG in AIA rat model even though DRG macrophages were increased compared to those in naïve rat (77). By contrast, DRG macrophage was markedly increased in the SNI model (Fig. 3 *I* and *J*), supporting that DRG macrophages are a causal mechanism of neuropathic pain but not arthritis pain. However, DRG macrophage recruitment in SNI does not require TRPM2. On the other hand, TRPM2 is indispensable for the recruitment of macrophages and neutrophils in injured sciatic nerves (Fig. 3 *I*–*K*). Overall, TRPM2 exerts both pro- (for lymphocytes) and anti-inflammatory effect, which may explain the variable effects of TRPM2 on inflammation.

Different effects of TRPM2 on inflammatory cells seem to be related to their different origins. It has been shown that increased DRG macrophage in SNI and synovial macrophage in rheumatoid arthritis arise from proliferation of tissue resident macrophages, whereas recruited nerve macrophages originate from infiltration of peripheral blood monocytes (38, 78), and this process is mediated by immune TRPM2 (Fig. 3*J*). It thus appears that immune TRPM2 is indispensable for recruitment of blood-derived infiltrating immune cells but not for the expansion of tissue-resident immune cells such as DRG macrophages and synovial resident macrophages. These resident immune cells are highly heterogenous and plastic primarily responsible for producing proinflammatory cytokines in response to “on-demand” signals contributing to chronic arthritis pain and neuropathic pain (35, 38, 78–80). However, deletion of neuronal TRPM2 affected neither DRG and nerve macrophages nor synovial macrophages in SNI and AIA models but markedly inhibited chronic neuropathic pain and arthritis pain. We thus concluded that neuronal TRPM2 transduces chronic pain independent of immune TRPM2 and inflammatory cells.

A notable finding in this research is that chronic arthritis pain was completely abolished by pharmacological blockade of TRPM2 channels in the knee joints but partially reduced by genetic deletion of TRPM2. Deletion of TRPM2 may activate unknown compensatory mechanisms leading to incomplete inhibitory effect. Nevertheless, the robust effect of TRPM2 inhibitor on chronic arthritis pain supports that TRPM2 is a highly promising drug target for the treatment of chronic arthritis pain, neuropathic pain, and potentially other chronic pain syndromes caused by autoantibodies and PGE2.

## Materials and Methods

Details are provided in *SI Appendix*. Briefly, all experiments were conducted in accordance with the UK Animals (Scientific Procedures) Act 1986. Antigen-induced arthritis pain and spare nerve injury neuropathic pain were generated in TRPM2 knockout and conditional knockout mice followed by pain behavior assessment. Inflammatory cell infiltration and joint pathology were determined using immunohistochemistry, H&E and Toluidine blue staining. Inflammatory cytokines, metabolites, PGE2, and IgG were quantified using ELISA and LC–MS/MS. Ca^2+^ imaging and electrophysiology were employed to determine neuronal responses to PGE2 and IgG-IC and TRPM2 activation. Significance between groups was determined using *student’s t* test and one-way ANOVA.

## Supplementary Material

Appendix 01 (PDF)

## Data Availability

All study data are included in the article and/or *SI Appendix*.
