# Supplementary material for: TRPM2 is a direct pain transducer
Source: Proc Natl Acad Sci U S A. 2026 Sep 16;123(38):e2532289123. doi: 10.1073/pnas.2532289123 (PMC13598259; doi:10.1073/pnas.2532289123)
Supplement: Supplementary file 1 [file pnas.2532289123.sapp.pdf]

PNAS

**Supporting Information for**

# TRPM2 is a Direct Pain Transducer


Linda Varghese[a,1], Mujahid Alizada[a,1], Jinquan Yang[a,1], Ye Feng[a], Xiaoqiu Yuan[a], Mitali Malhotra[a] and Xuming Zhang[a,2]

[a]School of Life Sciences, University of Warwick, Conventry CV4 7AL, United Kingdom

[1]L.V., M.A., and J.Y. contributed equally to this work

[2]To whom correspondence may be addressed. Email: xuming.zhang@warwick.ac.uk


**This PDF file includes:**

Supporting methods
Figures S1 to S8
SI References

## Supporting methods

### Animals

All the animal experiments were conducted in accordance with the project license granted by UK home office and approved by the animal welfare and ethical review committee at the University of Warwick. Animals were maintained in IVC cages in a 12h light/dark cycle with humidity and temperature centrally controlled and food and water available *ad libitum*. Age and sex-matched animals (>8 weeks) were randomly assigned to different groups, and experimenters were blinded to genotype.

TRPM2-knockout mice were obtained from Prof. Peter McNaughton (King's College London) permitted by Dr. Yasuo Mori (Kyoto University, Japan). TRPM2 floxed (strain no: 029826) and Advillin-Cre (strain no: 032536) mice were obtained from Jackson laboratory. The mice were backcrossed for at least 7 generations in C57/BL6 background. The genotype of transgenic mice was determined with PCR using genomic DNA isolated from ear tissue biopsy samples. Adult mice between the age of 8-16 weeks were used for all the experiments. Both male and female mice were used and assigned to experimental groups by random. The welfare of animals was regularly monitored throughout the experiments.

### Cell culture and transfection

HEK293 cells were cultured in DMEM high glucose (4.5g/L) medium supplemented with 2mM L-glutamine, 100U/ml penicillin, 100μg/ml streptomycin and 10% FBS (ThermoFisher) in a humidified incubator as described previously(1, 2). DRG neurons were isolated from adult mice and plated onto coverslips coated with poly-L-lysine (100μg/ml, Merck) as described previously(1). The neurons were briefly cultured in DMEM low glucose (1g/L) medium supplemented with and 1% FBS, 2mM L-glutamine, 100U/ml penicillin and 100μg/ml streptomycin and were used for $Ca^{2+}$ imaging and electrophysiology within 8 hours after isolation. Transfection of HEK293 cells with plasmid cDNA was conducted using TurboFect transfection reagent (ThermoFisher) as described previously(2). Transfected cells were used for electrophysiology and protein biochemistry analysis within 48h after transfection.

### Molecular Biology

hTRPM2 in pcDNA4/To vector was obtained from Dr. Andrew M. Scharenberg (University of Washigton, USA) and cloned into pCDNA3-V5-His (6x)-TOP vector (ThermoFisher) via NotI and XbaI sites. mTRPM2 in pCDNA3 clone was kindly provided by Prof. Peter McNaughton (King's College London). hFcγRI in pCMV3 vector with a Flag tag at the C-terminus was purchased from Sino Biological. All the G protein subunits including GαoA, GαoA Q205L, GαoB Q205L, Gαi1 Q204L, Gαi2 Q205L, Gαs Q227L and Gβ1γ2 and PGE2 receptors (EP1-EP4) were purchased from cDNA resource centre. Membrane targeted Mas-GRK2-ct binding to Gβγ devoid of kinase activity was a kind gift of Dr. Stephen R. Ikeda (NIH, USA)(3).

**Nickel beads pull down assay and Western blotting**

Pull down assay was used to probe interaction of TRPM2 with GαoA or FcγRI and performed as described previously(1, 2). Briefly**,** HEK293 cells co-expressing TRPM2-V5-His and GαoA or FcγRI-Flag were solubilised in lysis buffer containing 20mM Tris-HCl (pH.7.4), 300mM NaCl, 10% glycerol, 40mM imidazole, 0.2mM EDTA plus protease inhibitor cocktail (Merck). Cell lysate was then incubated with Ni-NTA beads (Qiagen) at 4°C overnight. Beads were then thoroughly washed in lysis buffer followed by boiling in sample buffer. Supernatant was then separated in 7.5%SDS-PAGE gel succeeded by protein transfer and detection using anti-GαoA (SC-13532, Santa Cruz) and anti-Flag (Genscript).

DRG and synovial tissues were isolated from mice and then solubilised in lysis buffer as described previously(1, 4). Total proteins were separated and transferred to PDVF blot followed by detection using anti-mouse IgG (1:5000, Jackson), anti-CD68 (1:2000, Biolegend) and anti-Ly6C/G (1:2000, Biolegend).

**Mouse pain models**

Spare nerve injury model was performed as described previously(5). Briefly, an incision was made on the skin in the mid-thigh of mice under anaesthesia using isoflurane. A blunt dissection was then made through the bicep femoris muscle to expose sciatic nerve and three distal branches: sural, common peroneal and tibial nerves. Common peroneal and tibial nerves were ligated using Vicryl suture (5-0) followed by transection distal to the ligation. Sural nerve was kept intact. Muscle and skin in the wound were closed with sutures.

Antigen-induced arthritis model was conducted as described by others(6). Animals were preimmunised (s.c.) with 200μg methylated bovine serum albumin (mBSA) (Merck) emulsified in 200μl Complete Freund's adjuvant (CFA) (Merck). Arthritis was then induced by intra-articular injection of 100μg mBSA into the right knee joints one week after immunisation. Arthritis pain was then assessed, and joint tissues were collected at different days after induction of arthritis.

Acute pain model induced by IgG-IC was conducted by intra-articular injection of 2μg IgG-IC in 10μl saline into the right knee joint. IgG-IC was prepared by incubating BSA (10μg/ml) (Merck) with anti-BSA (2mg/ml) (MP biomedicals) in 1:3 ratio in PBS at 37°C for 40min. Acute pain model induced by PGE2 was carried out by intraplantar injection of 1μg PGE2 (Tocris Bioscience) in 10μl saline.

**Behavioural assays**

**Automated Von Frey test:** Mechanical pain was measured using dynamic plantar aesthesiometer (Ugo Basile) as described previously(1, 4). Animals were acclimatised in a transparent enclosure (9.6 x 9.6 x 14cm) on a metal mesh for at least 30 min before experiments. Mechanical force was applied perpendicularly in a linear increase to the paws of mice using a standardised probe controlled by a stimulating motor. Threshold mechanical force at which a paw withdrawal response is elicited in animals was then automatically recorded by the device. Animals were tested for at least two trials with at least 5min apart between the trials. The advantage of automated Von Frey is constant, reproducible and standardised force application, reducing the variability in manual filament application.

**Hargreaves test:** thermal hyperalgesia was monitored using Hargreaves apparatus (Ugo Basile). After full habituation of animals, the hind paw of animals was exposed to an infrared heat source. Once animals feel pain and withdraw their paws, latency to paw withdrawal was automatically recorded.

**Acetone evaporation test:** A drop of acetone (50μl) was directly applied to the hind paws of animals using a syringe. The duration of animals spent on flinching, lifting, licking and biting the paws were recorded.

**Weight bearing test:** The habituated animals were placed on an Incapacitance Tester (Linton Instrumentation) with two hind paws sitting on the two weight sensors, respectively. Body weight borne by each hind limb was automatically recorded and weight distribution between two limbs were calculated.

**PAM test:** primary joint pain was measured using a Pressure Application Measurement (PAM) device (Ugo Basile). A force senor was directly applied to the joints of mice. Threshold force eliciting a limb withdrawal response was recorded by the device.

**Histology**

Animals were transcardially fixed by perfusion with PBS followed by 10% formalin solution under anaesthesia with isoflurane. Knee joints were collected and decalcified in 10% EDTA for 30 days followed by treatment with 30% sucrose. The lumbar L4-L5 DRGs, sciatic nerves, and skin samples were also collected and postfixed in 4% paraformaldehyde overnight followed by 30% sucrose overnight for cryopreservation. Tissues were then embedded in OCT compound and sectioned in a cryostat at 12μm. Tissue sections were loaded on poly-lysine coated slides for staining.

**Immunohistology**

Tissues sections were blocked and permeabilised in 5% donkey serum and 0.4% Triton X-100 at RT for 30min. They were then incubated at 4°C overnight with the following primary antibodies: TRPM2 (1:200, rabbit, Alomone lab), CD68 (1:100, mouse, 137002, Biolegend), CD3 (1:200, rat, eBioscience), Ly6C/G (1:200, rat,108412, Biolegend), Anti-mouse IgG (H+L) (1:500, goat, Jackson ImmunoResearch), TRPV1 (1:100, mouse, Santa Cruz), Substance P (1: 100, mouse, MAB4375, Bio-Techne), FITC-conjugated IB4 (1:100, L2895, Merck). After wash, sections were next incubated at RT for 2h with fluorescence-conjugated secondary antibodies (1:500, Alexa Fluor 488/594 donkey/chicken anti-rabbit/anti-mouse/anti-rat or Alexa Fluor 488 donkey anti-goat). The stained sections were mounted onto slides for examination using a Zeiss confocal microscopy. A minimum of five images per animal sample and at least 3 animals were obtained for each experimental group. All the images were analysed using Fiji Image J software.

**In situ hybridization (ISH)**

ISH for detection of TRPM2 mRNA combined with immunohistochemistry (IHC) for staining TRPV1 in DRG was performed as described previously(7). TRPV1 IHC was first conducted and imaged as described above followed by ISH. Anti-sense TRPM2 cRNA (468bp) was in vitro transcribed from mouse TRPM2 cDNA with T7 RNA polymerase and labelled using digoxigenin RNA labelling kit (Roche). Tissue sections were pretreated with protease K and blocked with hybridization blocking solution (Roche). They were then incubated with anti-sense TRPM2 RNA in the hybridization solution. Tissues were then thoroughly washed and incubated with alkaline phosphatase (AP)-conjugated antibody (1:1000; Roche) and 5-bromo-4-chloro-indolyl-phosphate/nitroblue–tetrazolium (BCIP/NBT) substrates for color development. The color images were converted to pseudo-fluorescent images and then merged with TRPV1 fluorescent images.

**Haematoxylin & Eosin (H&E) staining**

Tissue sections were stained in 0.1% Mayers haematoxylin at RT for 10min followed by washing with tap water. The tissues were then counterstained briefly with 0.01% Eosin solution and sealed by coverslip.

**Toluidine Blue staining**

Tissue sections were first briefly treated with 10% neutral buffered formalin solution and then washed in deionised water. They were then stained with 0.02% Toluidine Blue solution for 40 seconds. After dehydration, the tissues were sealed by coverslip using Vectamount solution. Proteoglycan loss, cartilage damage and erosion in the knee joints were quantified and scored based on the “SMASH” recommendations (0-3)(8).

**ELISA**

Joint tissues were collected from animals and snap frozen immediately in liquid nitrogen. The tissues were then homogenised in 1ml PBS containing Complete EDTA-free protease inhibitor cocktail (Merck) using a pestle and mortar. Protein concentration of tissue extracts was determined using Bradford reagent (Merck). The concentration of IL-1β, IL-6, TNFα, PGE2, substance P, CGRP and IgG in joint tissues were next determined using ELISA kits (Bio-techne) in accordance with the manufacturers’ instructions. Briefly, samples and standard in duplicate

were added with assay diluent to each well of a microplate followed by incubation at RT or 37°C (for CGRP) for 2-3h. Sample wells were then washed and incubated with mouse IL-1$\beta$/IL-6/TNF$\alpha$/PGE2/substance P conjugates at RT for 2-3h. Substrate solution was then added for colour development at RT for 15-30min. Tissue IgG was determined using a mouse IgG uncoated ELISA kit (Invitrogen) following manufacturer's instructions. The samples and standards were added to a microplate precoated with anti-mouse IgG antibody. After incubation and washing, IgG in the samples was detected with HRP-conjugated anti-mouse IgG antibody followed by colour development using substrate solution. Colour density in all the samples was finally determined using a microplate reader at 450nm.

**LC-MS/MS**

Lumbar DRG (L4-L5) and sciatic nerves were isolated from animals and then homogenised in a lysis buffer containing 100mM Tris-HCl (pH 7.4), 150mM NaCl, 1mM EDTA, 1mM EGTA plus protease inhibitor cocktail (Merck). Samples were then centrifuged at 1000rpm for 5min. The supernatants were then mixed with equal volume of cold methanol on ice for 20min followed by centrifuge at 14000rpm for 10min. The supernatants were then transferred to an Ultrafree centrifuge filter (Merck) and subjected to centrifuge at 11300rpm for 2min. The filtrate was then snap frozen for further analysis of ADPR and PGE2 by LC-MS. Briefly, samples were loaded onto a Waters Acquity HPLC HSS T3 1.8$\mu$m 2.1 x 150mm column and then separated with increasing gradient of acetonitrile solution. Eluted molecules were then positively ionised through heated electrospray ionization (H-ESI) followed by separation and detection in a Quantiva triple quadrupole mass spectrometer (Thermo Scientific). ADPR and PGE2 were identified by different retention time, unique mass-to-charge ratio (m/z) and collision energy. The concentrations of ADPR and PGE2 were calculated based on the area under peaks by comparing to a positive standard of ADPR/PGE2 using Skyline software (version 25.1).

**Electrophysiology**

Whole-cell patch clamping was performed on DRG neurons and HEK293 cells at room temperature using thin-walled patch pipettes with a resistance between 2-4$\Omega$. Signals were amplified using an Axopatch 200B amplifier and digitised using a Digidata 1440A (Molecular Device). Excitability of DRG neurons was assessed using current patch clamping with pipette solution containing (in mM) 140 KCl, 1 MgCl2, 5.0 EGTA, 2.5 MgATP, 0.5 Li GTP and 10 HEPES (pH

7.4 with KOH) and bath solution containing (in mM): 140 NaCl, 4 KCl, 10 HEPES, 1.8 CaCl2, 1 MgCl2, 5 Glucose (pH 7.4 with NaOH). The action potentials were elicited by injecting current steps with an increment of 10pA in 500ms before and after treatment of DRG neurons with PGE2 (10μM, 4min). TRPM2 currents were recorded using whole-cell voltage patch clamping with pipette solution containing (in mM): 140 KCl, 10 HEPES, 1 MgCl2, 2 EGTA (or BAPTA) (pH 7.4 with KOH). To determine ADPR and G protein signaling, 8-Br-ADPR (300μM, Biolog) or GDP-β-S or GTP-γ-S (Merck) was included in pipette solution indicated in the relevant figures. Bath solution was similar as above except that 1.8mM CaCl2 was replaced by 1mM CaCl2. Series resistance was compensated (>80%) and cells were held at -70mV. The I-V relationship of TRPM2 channels was examined by delivering 500ms duration of voltage ramps from -100mV to +100mV from a holding potential of 0mV. Data were analysed off-line using Clampfit 11.0 (Molecular device).

**Ratiometric $Ca^{2+}$ imaging**

$Ca^{2+}$ imaging was performed as described previously(1). Briefly, DRG neurons were loaded with 3.3μM Furo-2-AM (ThermoFisher) at 37°C for 20min. Neurons imaged using a Nikon Ti2 inverted microscope connected to a sCMOS camera (Photometrics) by exposing neurons alternatively to 340nm and 380 LED illuminators (Cairn Research UK). Emission signals were collected at 510nm every two seconds. Cells were continuously perfused with Hanks' balanced salt solution containing (in mM): 140NaCl, 4 KCl, 10 HEPES, 1.8 CaCl2, 1 MgCl2 and 5 Glucose (pH 7.4). Solution perfusion and drug application were controlled by an automated perfusion system (Warner Instruments). A 10% increase in F340/F380 over baseline was counted as a positive response.

**Statistical analysis**

All data are expressed as mean ± SEM. Significance between groups was determined using Student's *t* test or one way ANOVA when comparisons between multiple groups were required. Two tailored test was used in the study. Chi-squared test was performed for Fig. 3i and Fig. 4f. *P*<0.05 was considered to be significantly different. *, #, *P*<0.01; **, ##, *P*<0.01; ***, ###, **P**<0.001.

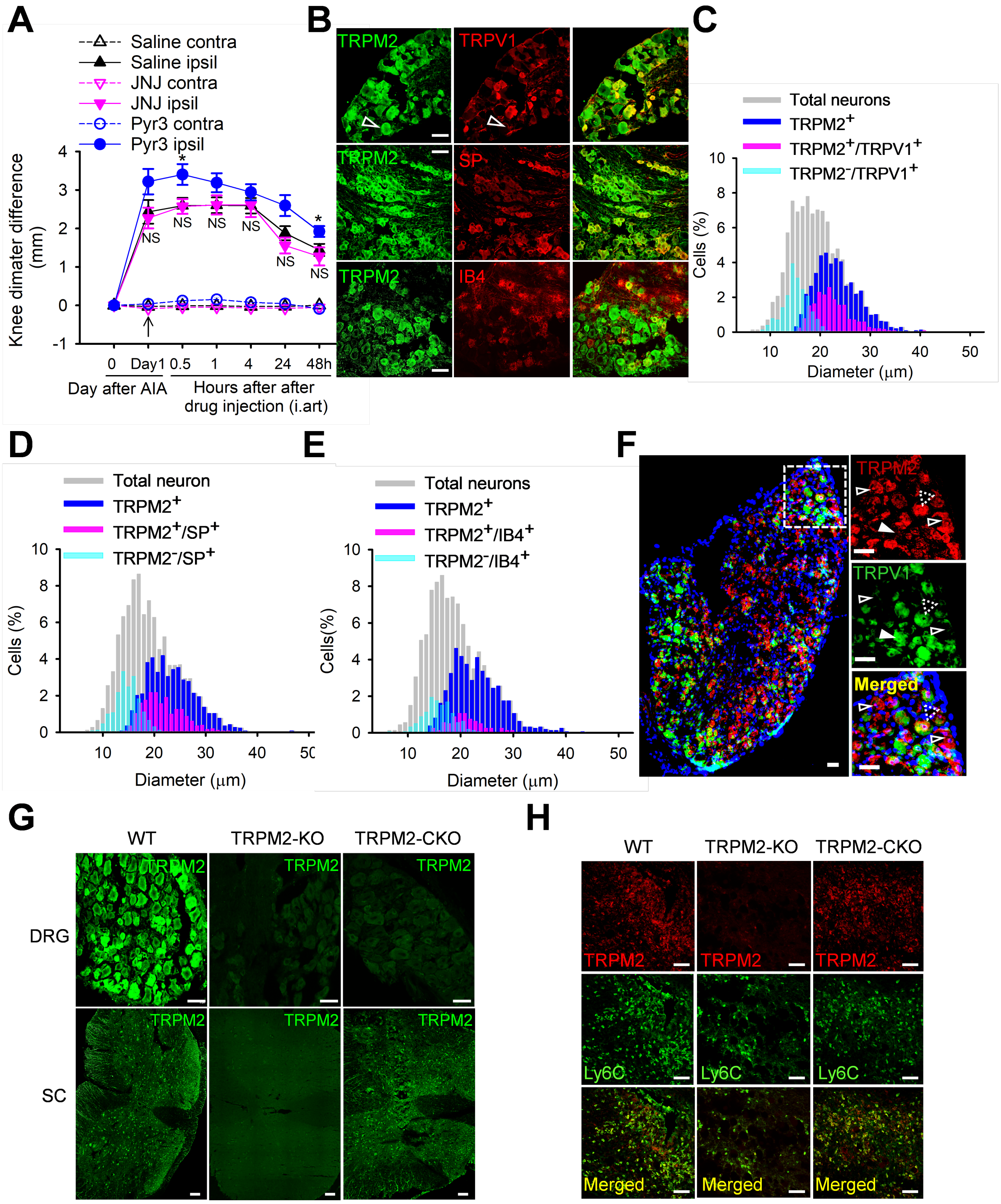


**Fig. S1. TRPM2 expression and deletion in sensory DRG neurons.** (**A**) Joint diameter difference in mice after AIA induction followed by intraarticular injection of saline, JNJ-28583113 (2mM) and Pyr3 (2mM). Arrow denotes drug administration (i.art). n=5-8 per group. (**B**) Example of immunostaining of TRPM2 with TRPV1(top panel), SP (middle panel) and IB4 (bottom panel) in lumbar DRG neurons. Empty arrowheads indicate $TRPM2^{+}/TRPV1^{-}$ neurons. Scale bars, 50μm. (**C-E**) Histogram distribution of $TRPM2^{+}$ DRG neurons with TRPV1-, SP- and IB4-expressing DRG neurons from experiments similar to those in (B). $n_{cell}$=2576 (**C**), $n_{cell}$=2628 (**D**), $n_{cell}$=1605 (**E**). (**F**) Co-expression of TRPM2 mRNA (detected with ISH) with TRPV1 (detected with immunohistochemistry) in lumbar DRG neurons. Empty

and solid arrowheads indicate $TRPM2^+/TRPV1^-$ and $TRPM2^-/TRPV1^+$ DRG neurons, respectively. Dotted empty arrowhead denotes $TRPM2^+/TRPV1^+$ neurons. Scale bars, 100μm (left panel), 40μm (right panels). (**G**) Immunohistochemistry staining of TRPM2 in the lumbar DRG and spinal cord (SC) from WT, TRMP2-KO and TRPM2-CKO mice. Scale bars, 50μm (top panels); 100μm (bottom panels). (**H**) Co-expression of TRPM2 with neutrophils/monocytes marker Ly6C in the synovium (bottom panels) of the knee joints from AIA mice (3 days after AIA) in WT, TRPM2-KO and TRPM2-CKO mice. TRPM2 expression is lost in the synovium of TRPM2-KO mice but present in the synovium of WT and TRPM2-CKO mice. Scale bars, 50μm.

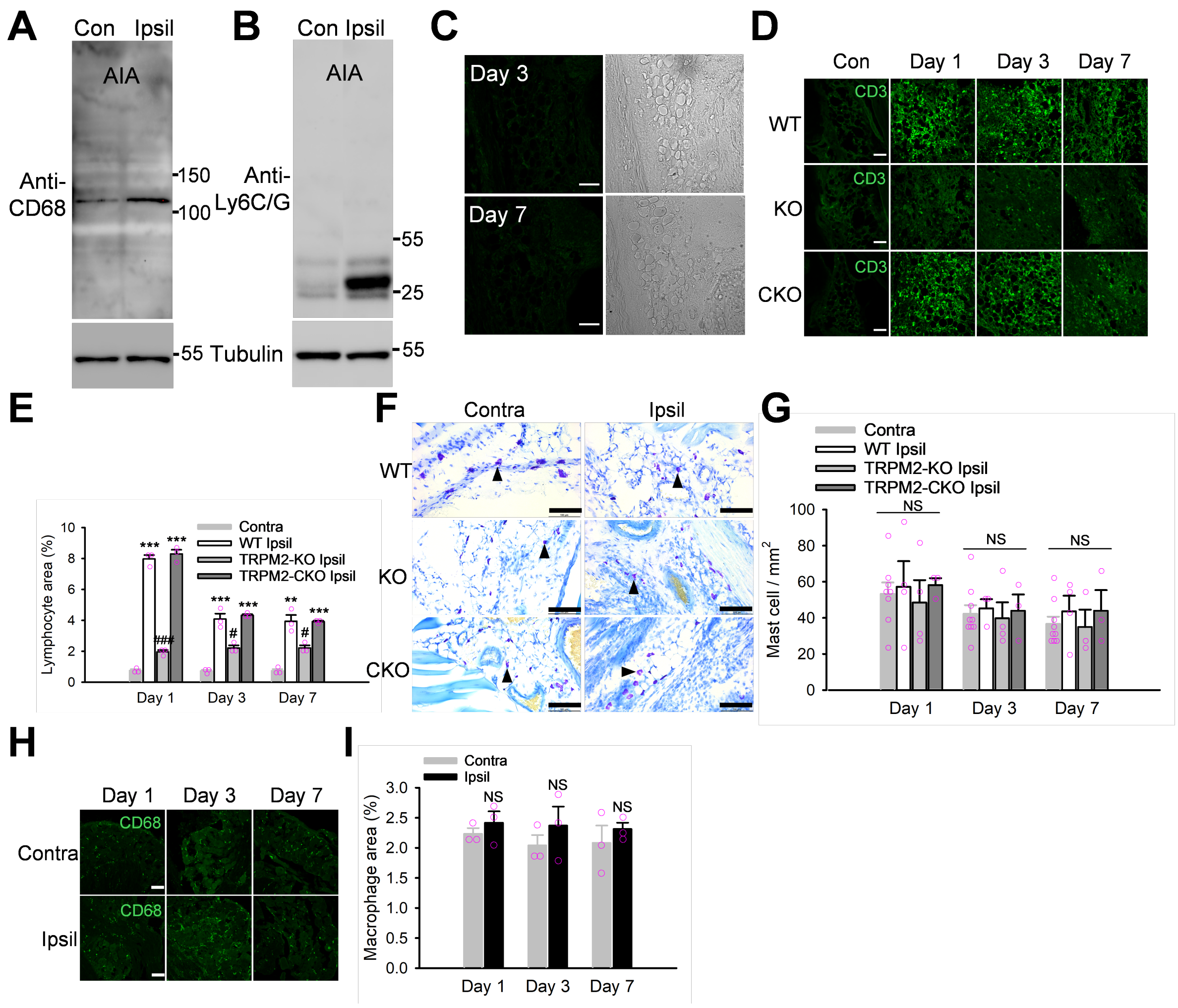


**Fig. S2. Neuronal TRPM2 is not involved in inflammatory cell recruitment in the AIA model.** (**A, B**) Expression of CD68 (**A**) and Ly6C/G (**B**) in the lysate of the contralateral (Con) and ipsilateral (Ipsil) knee joint tissues from mice after 3 days of AIA induction. The blots were redetected by anti-tubulin (bottom blots). (**C**) Immunohistochemistry staining of knee joints from WT mice after different days of AIA induction similar to those in Fig. 3A-3B but without primary antibodies. Transmission images are shown on the right indicating synovia hyperplasia. Scale bars, 50µm. (**D**) Example fluorescence images of lymphocytes stained by anti-CD3 in the synovial tissues of knee joints from WT, TRPM2-KO and -CKO mice at different days after AIA induction. Scale bars, 50µm. (**E**) Summary of percentage of lymphocyte-populated synovial areas from similar experiments to those in (**D**). *** compared to contra; #, ### compared to WT Ipsil. (**F**) Representative images of mast cells revealed by Toluidine blue staining in the contralateral and ipsilateral synovial tissues of knee joints from WT, TRPM2-KO and -CKO mice one day after AIA induction. Arrows indicate mast cells. Scale bars, 100µm. (**G**) Summary of quantification of mast cell density from similar experiments to those in (**F**). (**H**) DRG macrophages stained by anti-CD68 in the contralateral and ipsilateral DRG from mice after different days of AIA induction. Scale bars, 50µm. (**I**) Collective results of macrophage occupied synovial areas from similar experiments to those in (**H**). NS, not significant.

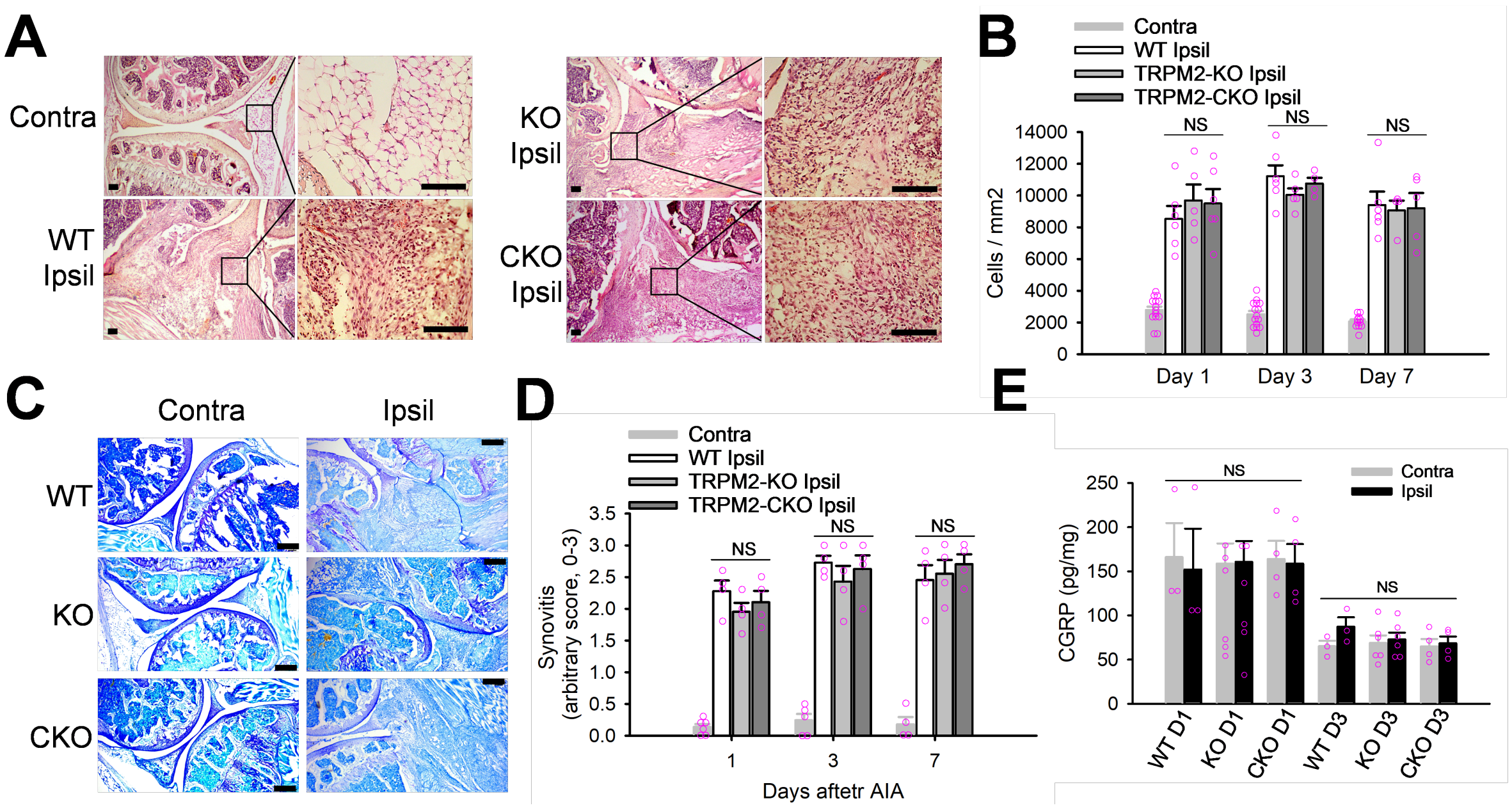


**Fig. S3. TRPM2 has no effect on joint pathology in AIA mice.** (**A)** Representative images of HE staining of the contralateral and ipsilateral knee joints from WT, TRPM2-KO and -CKO mice 3 days after AIA induction. Zoomed synovial tissues indicated by the squared box in the left image panels are shown on the right. Scale bars, 100μm. (**B)** Summary of quantification of cell density in the synovial tissues from similar experiments to those in (**A**). Deletion of TRPM2 does not affect synovial hyperplasia. NS, not significant. (**C)** Toluidine blue staining of the contralateral and ipsilateral knee joints from WT, TRPM2-KO and -CKO mice 7 days after AIA induction. Scale bars, 200μm. (**D)** Summary of scoring of cartilage damage and proteoglycan loss from similar experiments to those in (**C**). TRPM2 deletion does not affect joint pathology. NS, not significant. (**E)** CGRP concentrations in the contralateral and ipsilateral knee joints in AIA mice measured by ELISA.

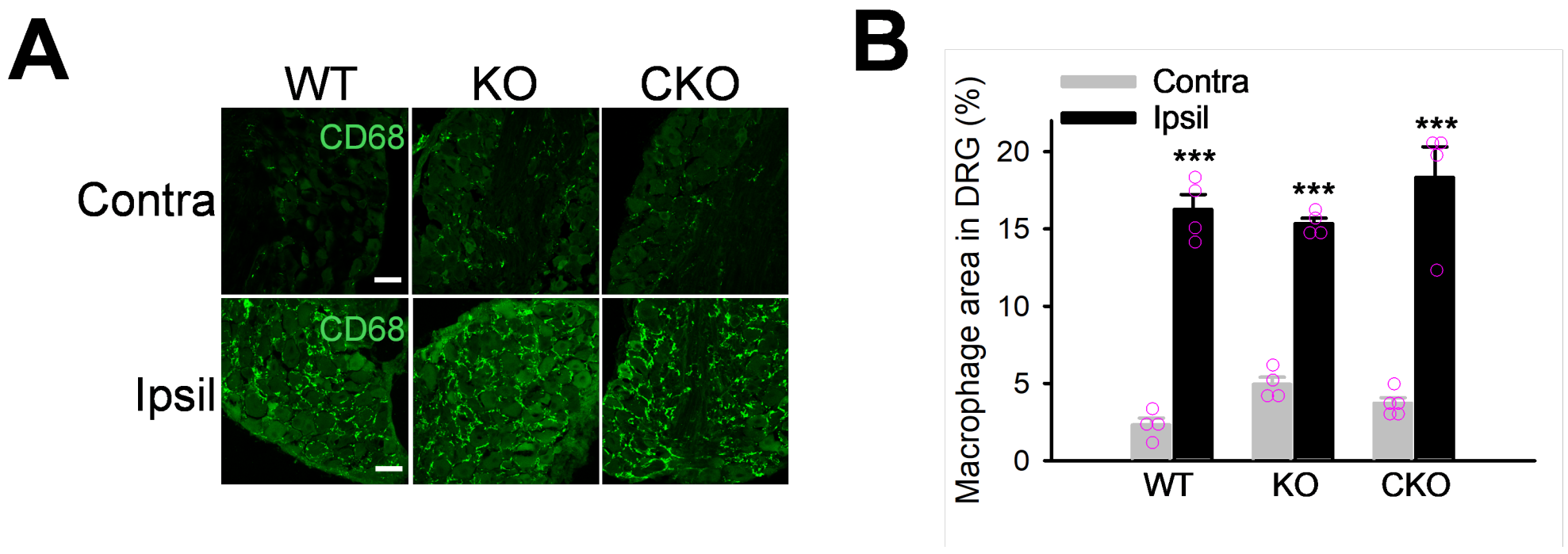


**Fig. S4. TRPM2 does not affect DRG macrophage recruitment in SNI model. (A** and **B)** Example images of DRG macrophages stained by anti-CD68 (**A**) and summary (**B**) of macrophage-occupied areas in the contralateral and ipsilateral lumbar DRG from SNI mice 7 days after nerve injury. Scale bars, 50μm.

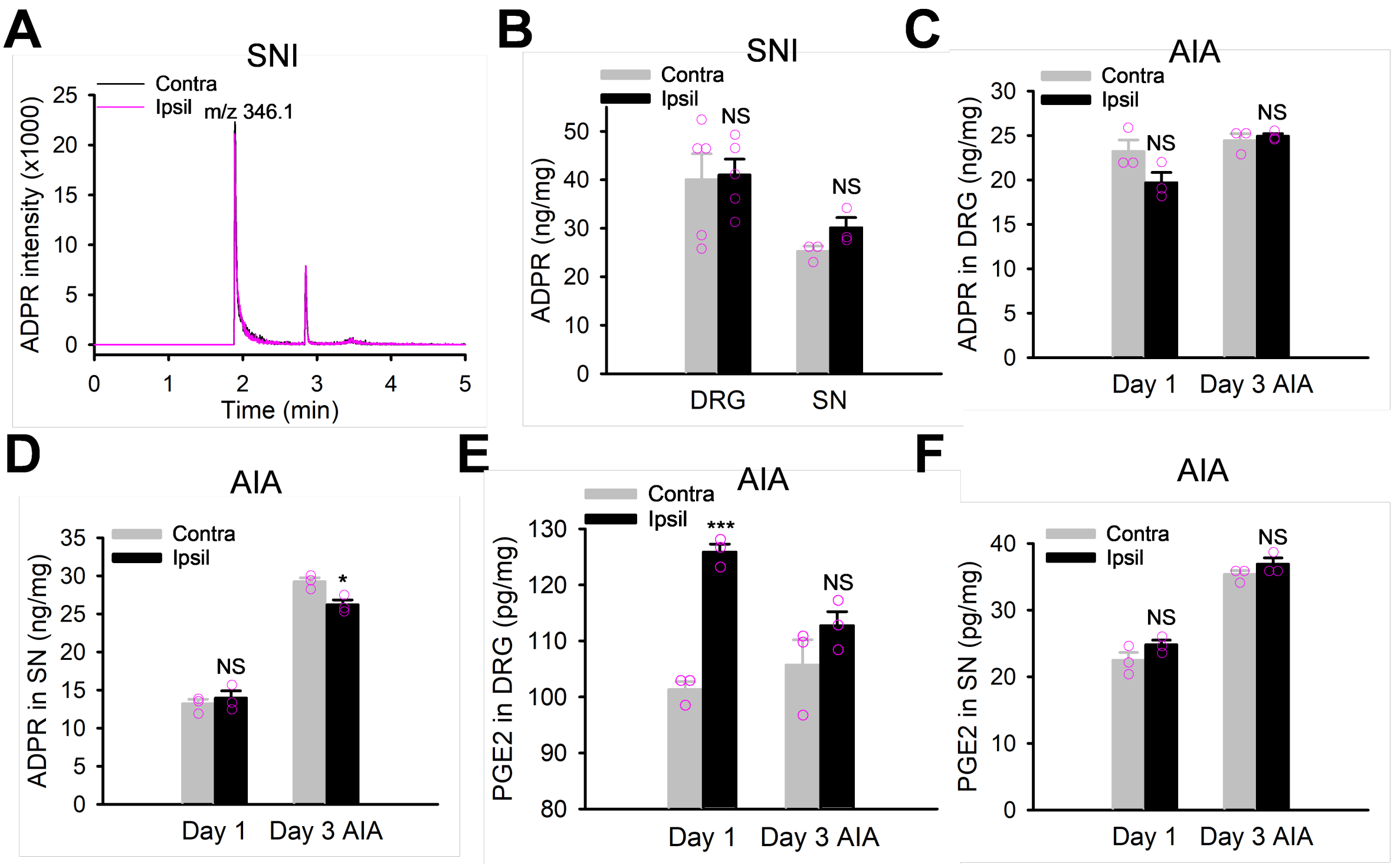


**Fig. S5. ADPR and PGE2 concentrations in the DRG and sciatic nerves (SN) of mice. (A**) Example ADPR peaks from the contralateral and ipsilateral DRG tissues from SNI mice detected by LC-MS. The peaks appear close to 2min after collision at an expected m/z ration of 346.1. (**B)** Summary of ADPR concentrations in the DRG and sciatic nerves (SN) from SNI mice seven days after nerve injury from similar experiments to those in (A). (**C** and **D)** ADPR concentrations in the contralateral and ipsilateral DRG (**C**) and sciatic nerves (SN) (**D**) from mice at Day 1 and Day 3 of post-AIA induction. NS, not significant. **P*<0.05. ADPR is even reduced in SN three days after AIA induction. (**E** and **F)** PGE2 concentration in the DRG (**E**) and SNs (**F**) from mice after different days of AIA induction measured using LC-MS similar to those in Fig. 3A. *** compared to the contralateral samples. NS, not significant.

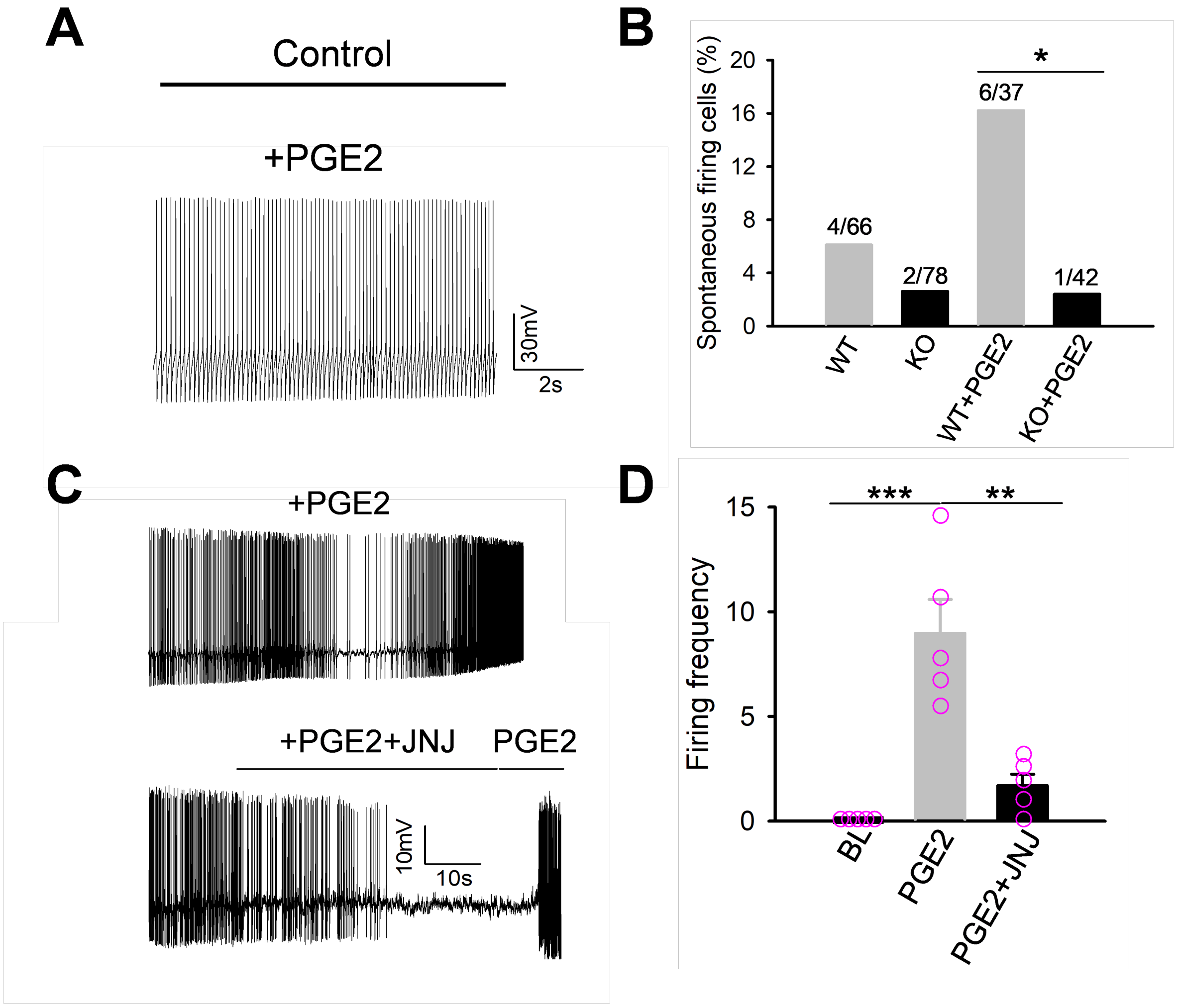


**Fig. S6. TRPM2 mediates spontaneous firing in DRG neurons evoked by PGE2. (A)** Example firing traces in a DRG neuron under control and after treatment with PGE2 (2μM, 3min). (**B)** Summary of the number of DRG neurons from WT and TRPM2-KO mice exhibiting spontaneous firing before and after treatment with PGE2 from experiments similar to those in (**A**). (**C)** Example firing of DRG neurons elicited by PGE2 (2μM, top panel) and in the presence of JNJ-28583113 (10μM) (bottom panel). After removal of JNJ-28583113, PGE2 elicited firing again. (**D)** Summary of firing frequency (spikes/s) in DRG neurons before and after treatment with PGE2 and JNJ28583113 from experiments similar to those in (**C**).

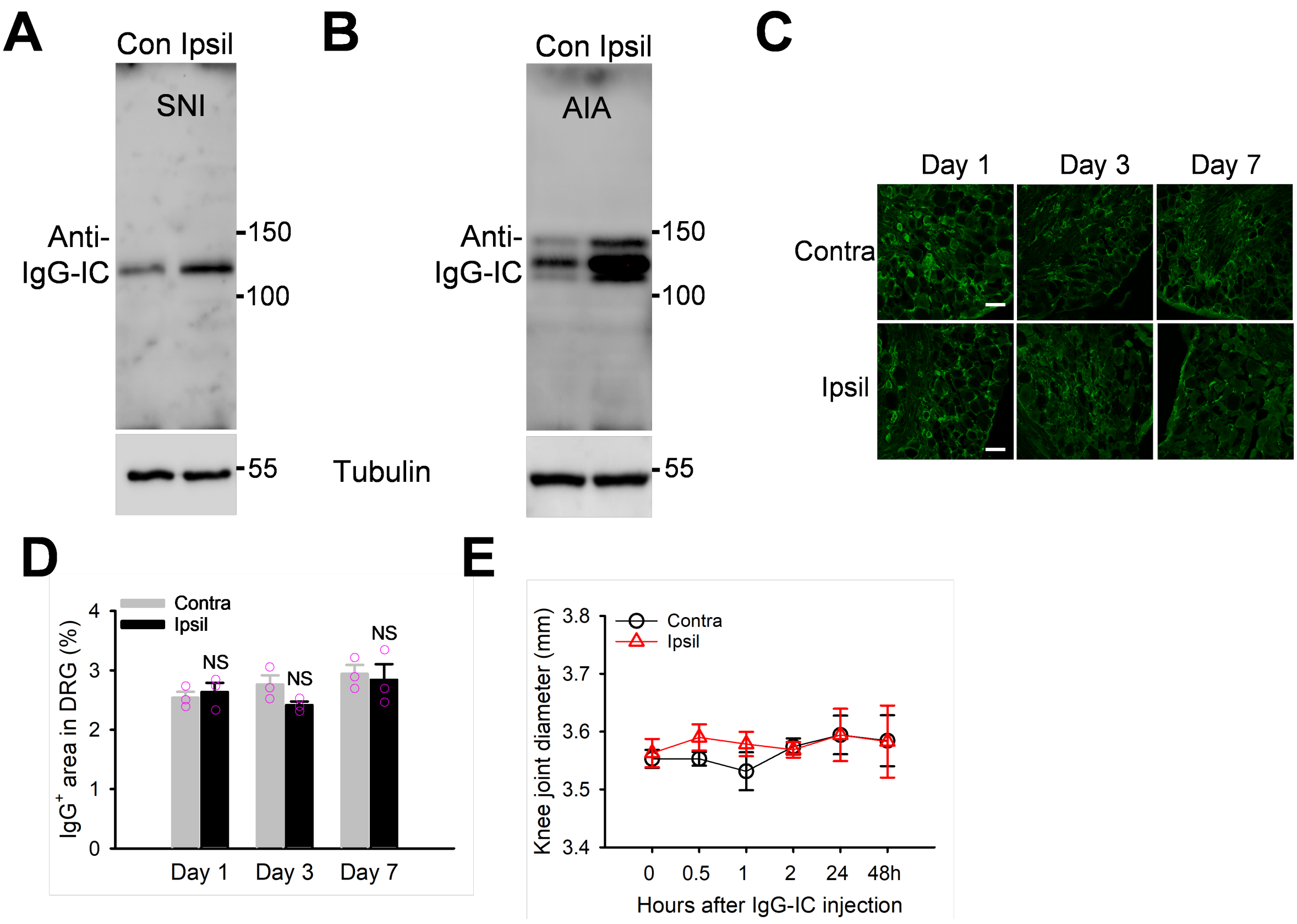


**Fig. S7. IgG is not increased in AIA model.** (**A, B**) IgG-IC expression in the contralateral (Con) and Ipsilateral (Ipsil) DRG from mice 7 days after spared nerve injury (SNI) (**A**) and knee joints from mice after 3 days of AIA induction (**B**). The blots were redetected by anti-tubulin (bottom blots). **(C)** Example fluorescence images of IgG immunoreactivity in the contralateral and ipsilateral DRG from AIA mice after different days of induction. Scale bars, 50µm. **(D)** Summary of IgG-positive areas in the DRG from similar experiments to those in (**C**). (**E)** Measurement of knee joint diameter at different time points after injection of IgG-IC (2µg). No significant joint inflammation is detectable. n=7.

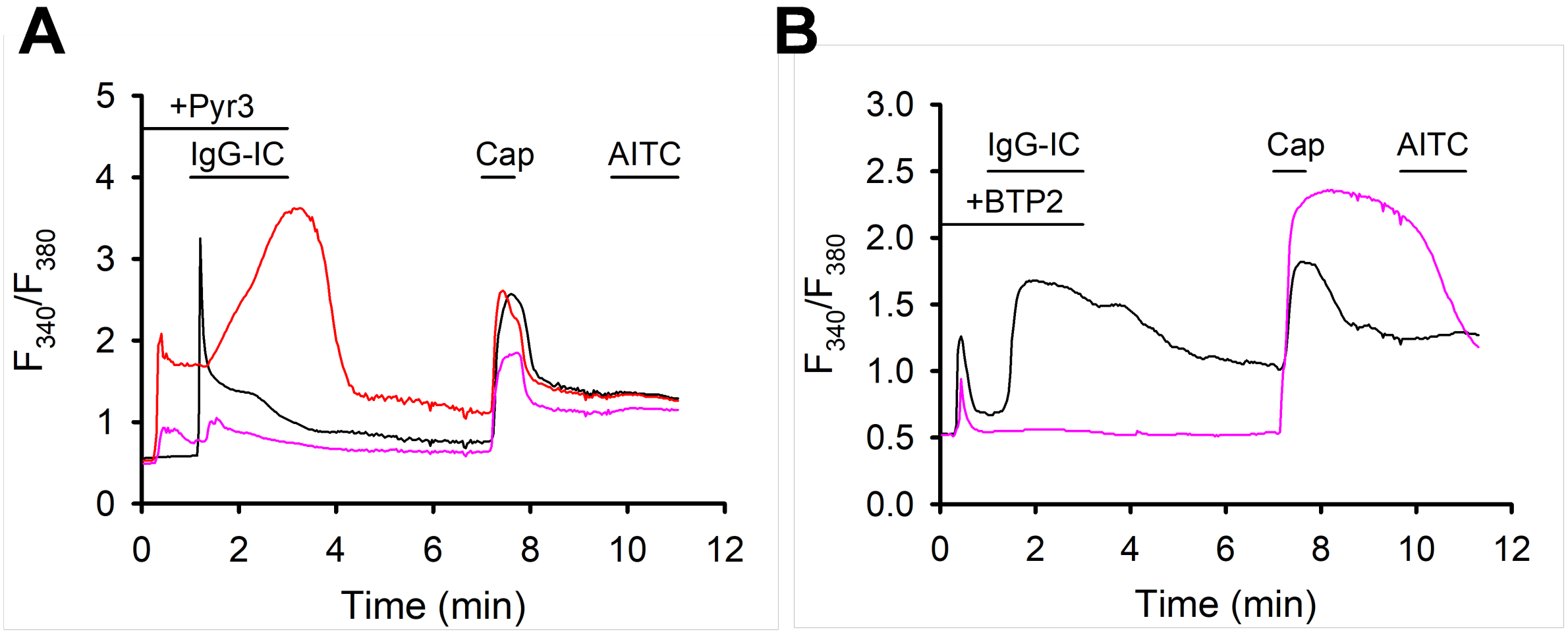


**Fig. S8. TRPC3 blockers elevate the basal [$Ca^{2+}$]$_i$ and enhance IgG-IC-induced $Ca^{2+}$ responses in DRG neurons. (A** and **B)** Example $Ca^{2+}$ responses in DRG neurons evoked by IgG-IC (1μg/ml) in the present of Pyr3 (10μM) (**A**) and BTP2 (10μM) (**B**). Both Pyr3 and BTP2 induced a basal $Ca^{2+}$ response and further enhanced IgG-IC-induced $Ca^{2+}$ responses.

## SNI references:

1. S. Khan *et al.*, IQGAP1 promotes chronic pain by regulating the trafficking and sensitization of TRPA1 channels. *Brain* **146**, 2595-2611 (2023).
2. X. Zhang, Direct Galphaq Gating Is the Sole Mechanism for TRPM8 Inhibition Caused by Bradykinin Receptor Activation. *Cell Rep* **27**, 3672-3683.e3674 (2019).
3. P. J. Kammermeier, S. R. Ikeda, Expression of RGS2 alters the coupling of metabotropic glutamate receptor 1a to M-type K+ and N-type Ca2+ channels. *Neuron* **22**, 819-829 (1999).
4. X. Zhang, P. H. Patra, J. Yang, Trafficking and potentiation of Nav1.7 and Nav1.8 channels are mediated by IQGAP1. *The Journal of physiology* 10.1113/jp290517 (2026).
5. I. Decosterd, C. J. Woolf, Spared nerve injury: an animal model of persistent peripheral neuropathic pain. *Pain* **87**, 149-158 (2000).
6. K. Midwood *et al.*, Tenascin-C is an endogenous activator of Toll-like receptor 4 that is essential for maintaining inflammation in arthritic joint disease. *Nature medicine* **15**, 774-780 (2009).
7. C. L. Chen *et al.*, Runx1 determines nociceptive sensory neuron phenotype and is required for thermal and neuropathic pain. *Neuron* **49**, 365-377 (2006).
8. S. Hayer *et al.*, 'SMASH' recommendations for standardised microscopic arthritis scoring of histological sections from inflammatory arthritis animal models. *Ann Rheum Dis* **80**, 714-726 (2021).
